# Diversity, evolution, and function of myriapod hemocyanins

**DOI:** 10.1186/s12862-018-1221-2

**Published:** 2018-07-05

**Authors:** Samantha Scherbaum, Nadja Hellmann, Rosa Fernández, Christian Pick, Thorsten Burmester

**Affiliations:** 1 Institute of Zoology, University of Hamburg, D-20146 Hamburg, Germany; 2 Institute for Biophysics, Johannes Gutenberg University of Mainz, D-55099 Mainz, Germany; 3 Museum of Comparative Zoology & Department of Organismic and Evolutionary Biology, Harvard University, 26 Oxford Street, Cambridge, MA 02138 USA; 4 Bioinformatics & Genomics Unit, Center for Genomic Regulation, 08004 Barcelona, Spain

**Keywords:** Arthropoda, Evolution, Hemocyanin, Myriapoda, Phenoloxidase, Subunit diversity

## Abstract

**Background:**

Hemocyanin transports O_2_ in the hemolymph of many arthropod species. Such respiratory proteins have long been considered unnecessary in Myriapoda. As a result, the presence of hemocyanin in Myriapoda has long been overlooked. We analyzed transcriptome and genome sequences from all major myriapod taxa – Chilopoda, Diplopoda, Symphyla, and Pauropoda – with the aim of identifying hemocyanin-like proteins.

**Results:**

We investigated the genomes and transcriptomes of 56 myriapod species and identified 46 novel full-length hemocyanin subunit sequences in 20 species of Chilopoda, Diplopoda, and Symphyla, but not Pauropoda. We found in *Cleidogona* sp. (Diplopoda, Chordeumatida) a hemocyanin-like sequence with mutated copper-binding centers, which cannot bind O_2_. An RNA-seq approach showed markedly different hemocyanin mRNA levels from ~ 6 to 25,000 reads per kilobase per million reads. To evaluate the contribution of hemocyanin to O_2_ transport, we specifically studied the hemocyanin of the centipede *Scolopendra dehaani.* This species harbors two distinct hemocyanin subunits with low expression levels. We showed cooperative O_2_ binding in the *S. dehaani* hemolymph, indicating that hemocyanin supports O_2_ transport even at low concentration. Further, we demonstrated that hemocyanin is > 1500-fold more highly expressed in the fertilized egg than in the adult.

**Conclusion:**

Hemocyanin was most likely the respiratory protein in the myriapod stem-lineage, but multiple taxa may have independently lost hemocyanin and thus the ability of efficient O_2_ transport. In myriapods, hemocyanin is much more widespread than initially appreciated. Some myriapods express hemocyanin only at low levels, which are, nevertheless, sufficient for O_2_ supply. Notably, also in myriapods, a non-respiratory protein similar to insect storage hexamerins evolved from the hemocyanin.

**Electronic supplementary material:**

The online version of this article (10.1186/s12862-018-1221-2) contains supplementary material, which is available to authorized users.

## Background

Oxygen (O_2_) is required for efficient generation of metabolic energy. In many animals, O_2_ is transported in the body fluid (blood or hemolymph) by specific binding-proteins. Such respiratory proteins have long been considered unnecessary in Myriapoda, which appeared to be well equipped for diffusive O_2_ transport by the tracheal system [1]. In the past years, it has become evident that at least some myriapods possess hemocyanin (Hc) for O_2_ transport [2, 3].

Hc is the respiratory copper-protein typically found in many arthropod and molluscan species [4–10]. Molluscan and arthropod Hcs do not share significant sequence similarities and are most likely the result of convergent evolution from different types of tyrosinases [7, 11]. Arthropod Hcs form hexamers or oligohexamers (up to 8 x 6mers), which are composed of similar or identical subunits of ~ 75 kDa [4–7, 9]. In each subunit, O_2_ binds to a pair of Cu^+^-ions, which are coordinated by six histidine residues of the protein chain (type III copper-center). In addition to its prominent role in O_2_ -transport and -storage, arthropod Hc may carry out other functions, such as a source for antimicrobial peptides [12], as phenoloxidase [13] or as storage protein that provides amino acids and metabolic energy [14].

Arthropod Hcs are members of a protein superfamily that additionally includes arthropod phenoloxidases (POs), which are copper-containing enzymes involved in various immunological functions and cuticle building [10, 15], and various proteins that are involved in energy and amino acid storage, such as pseudo-hemocyanins (cryptocyanins) of some decapod crustaceans, hexapod hexamerins, and dipteran hexamerin receptors [6, 9, 10, 15–18]. Sequences that share significant similarities with the arthropod proteins have been found in tunicates, hemichordates, sponges, and fungi [19–21]. Although functional analyses are missing, these proteins probably act as phenoloxidases.

Hc structure, function, and subunit evolution have been thoroughly studied in Chelicerata and Crustacea [5–7, 10, 22–26]. In the past about 15 years, Hc has also been identified in Hexapoda [27, 28] as well as in Onychophora [29], which are the sister taxon of the (eu-)arthropods. The first evidence for the presence of Hc in Myriapoda was found in the centipede *Scutigera longicornis* (Scutigeromorpha, Chilopoda) [30]. Mangum and colleagues [3] demonstrated that the Hc from *Scutigera coleoptrata* is a 6×6-mer, which is composed of four distinct subunit types [31, 32]. The presence of Hc in *Scutigera* was attributed to the high activity of this species and its blind-ending tracheal system [3]. However, a structurally similar Hc, which occurs as a mixture of 6×6-mers and 3×6-mers, was identified in the hemolymph of *Spirostreptus* sp. (Spirostreptida, Diplopoda) [2] and *Archispirostreptus gigas* [33]. In *Polydesmus angustus* (Polydesmida, Diplopoda) Hc occurs as a 3×6-mer [34].

Here, we present a thorough survey of Hc in 56 myriapod species, which has been made possible by the recent availability of transcriptome and genome data [35–39]. Employing an RNA-seq approach, we found large differences in mRNA levels across species. By specifically analyzing the Hc of the centipede *Scolopendra dehaani*, we demonstrated that Hc can contribute to O_2_ transport in the hemolymph even at low expression levels and that Hc may have a specific role in early myriapod development.

## Methods

### Databases and hemocyanin sequences

Short read data (Illumina or 454) of transcriptomes from 54 myriapod species were obtained from the SRA database at NCBI (https://www.ncbi.nlm.nih.gov/sra/) (Table 1). The transcriptomes were assembled with Trinity [40] using the standard settings. Contigs with > = 500 bp were kept and searched for *Hc* and *PO* cDNA sequences using a locally installed BLAST tool [41]. The derived sequences were verified by back-mapping of the reads from the corresponding species employing the CLC Genomics Workbench 11.0.0 (https://www.qiagenbioinformatics.com/). This approach was also used to extend partial sequences. Few incomplete or ambiguous Hc sequences that could not be resolved were discarded. The genome of *Strigamia maritima* Leach, 1817 [38] was searched and analyzed for Hc and PPO sequences at http://www.ensembl.org/. Genomic sequences of *Trigoniulus corallinus* were obtained from [39]. Scaffolds and contigs that cover the single *Hc* gene were assembled by hand. The *Hc* coding sequences were predicted by AUGUSTUS (http://bioinf.uni-greifswald.de/webaugustus/) [42] and GENSCAN (http://genes.mit.edu/GENSCAN.html) [43], and verified by the aid of a multiple sequence alignment with other myriapod Hc sequences. The cDNA sequences were translated into proteins with the translate tool at the ExPASy Server of the Swiss Institute of Bioinformatics (http://web.expasy.org/translate/).

**Table 1:** List of short read sequences used in this study

Species	Order	Class	SRA	Tissue	Sequencing	Hemocyanin	RPKM
*Hanseniella sp.*		Symphyla	SRX1734405, SRX3326582	whole body, body tissue	Illumina	1 Hc	328.1
*Scutigerella* sp.		Symphyla	SRX1734406	whole body	Illumina	1 Hc	997.7
*Symphylella vulgaris*		Symphyla	SRX246919	whole body	454	n.d.	0
*Symphylella sp.*		Symphyla	SRX3256428	trunk	Illumina	n.d.	0
*Pauropus huxleyi*		Pauropoda	SRX3257465	whole body	Illumina	n.d.	0
*Eudigraphis taiwaniensis*	Polyxenida	Diplopoda	SRX1734393	body tissue	Illumina	n.d.	0
*Polyxenus lagurus*	Polyxenida	Diplopoda	SRX390592	whole body	454	n.d.	0
*Cyliosoma* sp	Sphaerotheriida	Diplopoda	SRX1734403	body tissue	Illumina	n.d.	0
*Glomeris marginata*	Glomerida	Diplopoda	SRX1638914	body tissue	Illumina	n.d.	0
*Glomeris pustulata*	Glomerida	Diplopoda	SRX246920	whole body	454	n.d.	0
*Glomeridesmus sp.*	Glomeridesmida	Diplopoda	SRX326775	n.a.	Illumina	n.d.	0
*Brachycybe lecontii*	Playtdesmida	Diplopoda	SRX326776	n.a.	Illumina	n.d.	0
*Petaserpes sp.*	Polyzoniida	Diplopoda	SRX326777	n.a.	Illumina	n.d.	0
*Cleidogona sp.*	Chordeumatida	Diplopoda	SRX326780	n.a.	Illumina	5 Hc	549.3
*Abacion magnum*	Callipodida	Diplopoda	SRX326781	n.a.	Illumina	4 Hc	232.2
*Chamberlinius hualienensis*	Polydesmida	Diplopoda	DRX028808	animals without gut	454	3 Hc	2145.4
*Polydesmus angustus*	Polydesmida	Diplopoda	SRX390267	whole body	454	3 Hc	195.8
*Pseudopolydesmus sp.*	Polydesmida	Diplopoda	SRX326779	n.a.	Illumina	3 Hc	460.6
*Prostemmiulus sp.*	Stemmiulida	Diplopoda	SRX326782	n.a.	Illumina	4 Hc	25,587.7
*Narceus americanus*	Spirobolida	Diplopoda	SRX1638916	body tissue	Illumina	n.d.	0
*Trigoniulus corallinus*	Spirobolida	Diplopoda	SRX700727	whole organism	Illumina (gemome)	1 Hc	n.a.
*Cambala annulata*	Spirostreptida	Diplopoda	SRX326783	n.a.	Illumina	4 Hc	1183.5
*Cylindroiulus punctatus*	Julida	Diplopoda	SRX1734404	body tissue	Illumina	n.d.	0
*Scutigera coleoptrata*	Scutigeromorpha	Chilopoda	SRX462011	n.a.	Illumina	5 Hc	22,719.8
*Scutigerina weberi*	Scutigeromorpha	Chilopoda	SRX1637773	body tissue	Illumina	4 Hc	1904.3
*Sphendononema guildingii*	Scutigeromorpha	Chilopoda	SRX1637754	body tissue	Illumina	4 Hc	3238.3
*Thereuopoda* cf. *longicornis*	Scutigeromorpha	Chilopoda	SRX450808	venom gland	454	n.d.	0
*Craterostigmus tasmanianus*	Craterostigmomorpha	Chilopoda	SRX461877	n.a.	Illumina	n.d.	0
*Craterostigmus crabilli*	Craterostigmomorpha	Chilopoda	SRX1638413	body tissue	Illumina	n.d.	0
*Anopsobius giribeti*	Lithobiomorpha	Chilopoda	SRX1638351	body tissue	Illumina	n.d.	0
*Eupolybothrus cavernicolus*	Lithobiomorpha	Chilopoda	ERX311347	whole organism	Illumina	n.d.	0
*Lithobius forficatus*	Lithobiomorpha	Chilopoda	SRX462145, SRX270896	n.a., whole organism	Illumina + 454	n.d.	0
*Lithobius sp.*	Lithobiomorpha	Chilopoda	SRX326784	n.a.	Illumina	n.d.	0
*Paralamyctes validus*	Lithobiomorpha	Chilopoda	SRX1638281	body tissue	Illumina	n.d.	0
*Notiphilides grandis*	Geophilomorpha	Chilopoda	SRX1734365	body tissue	Illumina	n.d.	0
*Henia brevis*	Geophilomorpha	Chilopoda	SRX1734368	body tissue	Illumina	n.d.	0
*Himantarium gabrielis*	Geophilomorpha	Chilopoda	SRX461787	n.a.	Illumina	n.d.	0
*Hydroschendyla submarina*	Geophilomorpha	Chilopoda	SRX1638908	body tissue	Illumina	n.d.	0
*Mecistocephalus guildingii*	Geophilomorpha	Chilopoda	SRX1638910	body tissue	Illumina	n.d.	0
*Stenotaenia linearis*	Geophilomorpha	Chilopoda	SRX1638912	body tissue	Illumina	n.d.	0
*Strigamia maritima*	Geophilomorpha	Chilopoda	SRX530370-SRX530372	mixed	Illumina (gemome)	n.d.	0
*Tygarrup javanicus*	Geophilomorpha	Chilopoda	SRX1638906	body tissue	Illumina	n.d.	0
*Akymnopellis chilensis*	Scolopendromorpha	Chilopoda	SRX1638823	body tissue	Illumina	n.d.	0
*Alipes grandidieri*	Scolopendromorpha	Chilopoda	SRX205685	n.a.	Illumina	2 Hc	19.6
*Cormocephalus westwoodi*	Scolopendromorpha	Chilopoda	SRX273033	venom gland	454	n.d.	0
*Cryptops hortensis*	Scolopendromorpha	Chilopoda	SRX457664	n.a.	Illumina	1 Hc	338.0
*Ethmostigmus rubripes*	Scolopendromorpha	Chilopoda	SRX275322, SRX423979, SRX272980, SRX275340	venom gland + epidermis	454	n.d.	0
*Newportia adisi*	Scolopendromorpha	Chilopoda	SRX1638658	body tissue	Illumina	3 Hc	573.4
*Rhysida longipes*	Scolopendromorpha	Chilopoda	SRX1638865	body tissue	Illumina	n.d.	0
*Scolopendra alternans*	Scolopendromorpha	Chilopoda	SRX275341	venom gland	454	n.d.	0
*Scolopendra morsitans*	Scolopendromorpha	Chilopoda	SRX273031	venom gland	454	n.d.	0
*Scolopendra mutilans*	Scolopendromorpha	Chilopoda	SRX286707, SRX286708	whole organism	Illumina	3 Hc	589.7
*Scolopendropsis bahiensis*	Scolopendromorpha	Chilopoda	SRX1734364	body tissue	body tissue	2 Hc	273.2
*Scolopocryptops sexspinosus*	Scolopendromorpha	Chilopoda	SRX1638758	body tissue	Illumina	fragments	n.a.
*Scolopendra dehaani*	Scolopendromorpha	Chilopoda	SRX390596	body tissue (trunk)	454	2 Hc	74.1
*Theatops spinicaudus*	Scolopendromorpha	Chilopoda	SRX1734363	body tissue	Illumina	1 Hc	6

*n.a.* not available, *n.d.* not detected, *RPKM* cumulative RPKM

### Cloning and sequencing of *S. dehaani* hemocyanins and phenoloxidase cDNAs

An adult female centipede *S. dehaani* Brandt, 1840 (Scolopendromorpha, Chilopoda) was obtained from a local pet shop. Selected tissues of *S. dehaani* were dissected, shock-frozen in liquid N_2_ and kept at − 80 °C until use. Total RNA was extracted either according to the method by Holmes and Bonner [44] or with the RNeasy Kit (Qiagen, Hilden, Germany) according to the manufacturers’ instructions. The quality of the RNA was checked by measuring the OD 260/280 ratio and by gel electrophoresis. cDNA was obtained using the SuperScriptTM III RNase H-Reverse Transcriptase Kit (Invitrogen, Karlsruhe, Germany), employing an oligo(dT)-primer. Partial *S. dehaani* Hc and PO sequences were obtained with PCR using sets of primers that had been generated on the basis of partial cDNAs [35] (Additional file 1: Table S1). Missing 5′ and 3′ ends were completed by the RACE technique using the GeneRacer™ kit (Invitrogen) according to the manufacturer’s instructions. The PCR products were cloned into the pGEM-T vector (Promega, Mannheim, Germany) and sequenced by a commercial service (GATC, Konstanz, Germany).

### Sequence analyses and phylogenetic studies

N-terminal signal sequences required for export into the extracellular space were predicted with SignalP 4.1 [45]. An alignment of the amino acid sequences of myriapod Hcs and prophenoloxidases (PPOs) (Additional file 2: Table S2) was constructed with MAFFT 7 [46] with the G-INS-i method and the BLOSUM 62 matrix. The final alignment covered 57 Hc and 16 PPO sequences and 868 characters (Additional file 3: Figure S1). An additional alignment was generated by eliminating poorly aligned sections by Gblocks v0.91b [47], employing the options for a less stringent selection (smaller final blocks, gap positions within the final blocks, less strict flanking positions). This approach resulted in an alignment of 531 amino acids. The best-fitting models of amino acid sequence evolution, LG [48] and WAG [49], were selected with ProtTest [50] under the Akaike Information Criterion. MrBayes 3.2.6 [51] was used for Bayesian phylogenetic analysis. The LG model was coded as GTR model with fixed priors using the prset command of MrBayes by specifying the parameters aarevmatpr and statefreqpr. Metropolis-coupled Markov chain Monte Carlo sampling was performed with one cold and three heated chains in two independent runs for 5 million generations on the CIPRES web portal [52]. Prior probabilities were equal, starting trees were random, and tree sampling was performed every 1000th generation. The final average standard deviation of split frequencies was < 0.005, suggesting convergence of the chains. Posterior probability densities of the nodes were estimated after discarding the initial 25% of the trees as burnin.

### Quantitative real-time reverse transcription-PCR

Reverse transcription was performed with 775 ng total RNA from eggs and fat body of *S. dehaani* employing the SuperScriptTM III RNase H-Reverse Transcriptase Kit (Invitrogen) and oligo(dT)20 primer according to the manufacturer’s instructions. Quantitative real-time reverse transcription PCR (qRT-PCR) was performed with primer sets specific for Hc subunits, PO, β-actin and RPLP0 (Additional file 1: Table S1) using the Power SYBR Green PCR Master Mix and the 7500 Real-Time PCR System (Applied Biosystems, Darmstadt). qRT-PCR reactions were performed in technical triplicates. Amplification was carried out using a standard PCR protocol (95 °C for 15 s, 58 °C for 15 s, and 72 °C for 30 s; 40 cycles) and fluorescence was measured at the last step of each cycle. The specificity of the amplification reactions was validated by analysis of the dissociation curve. The mRNA copy numbers were calculated with the standard curve approach, which employs a dilution series with plasmids carrying the respective cDNA sequences [53]. Calculations were performed with the 7500 Software 2.0.6 (Applied Biosystems).

### RNA-Seq

RNA-Seq analyses were performed with the CLC Genomics Workbench. The reads of the individual transcriptomes were mapped to the Hc cDNA sequences. The following parameters were applied: Masking mode = No masking, Update contigs = No, Match score = 1, Mismatch cost = 2, Cost of insertions and deletions = Linear gap cost, Insertion cost = 3, Deletion cost = 3, Length fraction = 0.5, Similarity fraction = 0.95, Global alignment = No, Auto-detect paired distances = Yes, Non-specific match handling = Map randomly. The mRNA levels of the Hc subunit were calculated as RPKM (Reads Per Kilobase exon model per Million reads).

### SDS-PAGE and western blotting

Proteins concentrations from total hemolymph, egg and fat body extracts were determined photometrically. The proteins were separated by SDS-PAGE on a 10% gel with standard conditions [54]. The gels were stained with 0.1% Coomassie Brilliant Blue dissolved in 10% acetic acid/ 25% isopropanol. For Western blotting, proteins were transferred onto nitrocellulose. Non-specific binding sites were blocked with 4% non-fat dry milk in TBS (20 mM Tris-HCl, pH 7.5, 150 mM NaCl). An antiserum raised in rabbits against *S. coleoptrata* Hc [32] was diluted 1:10,000 in 5% non-fat dry milk in TBS and used for detection overnight at 4 °C. After four successive washing steps with 0.1% Tween-20 in TBS, the secondary antibody (goat α-rabbit Fab; Dianova, Hamburg) was applied in a 1:10,000 dilution for 1 h at room temperature. After four additional washing steps, the Hc bands were detected with nitroblue tetrazolium and 5-Bromo-4-chloro-3-indolyl phosphate in 100 mM Tris-HCl, pH 9.5, 100 mM NaCl, in the dark. The intensity of the Hc bands was estimated with the ImageJ program (https://imagej.nih.gov/ij/).

### Oxygen binding curves

Oxygen-binding curves were determined by the polarographic-fluorometric method [55], which bases on the fluorescence of deoxygenated Hc upon excitation with light with a wavelength of 290 nm. The intensity of this fluorescence linearly decreases with increasing O_2_ saturation of Hc. The fluorescence was measured with Hitachi F4500 (Binninger Analytic, Germany) at 338 nm, while the oxygen concentration of the Hc solution was determined simultaneously with an oxygen electrode (Microelectrodes. Inc., Bedford, USA) equipped with a home-built amplifier. Hemolymph was diluted twofold with 100 mM Tris-HCl, 20 mM MgCl_2_, 20 mM CaCl_2_ directly after sampling. The diluted hemolymph was centrifuged (10 min, 12,000 x g) and the supernatant was further diluted into Ringer solution (500 mM NaCl, 12 mM KCl, 12 mM CaCl_2_, 20 mM MgCl_2_, 10 mM Tris/HCl) at the indicated pH. The resulting O_2_ binding curves were used to determine the half-saturation pressure P_50_ and, after conversion by a Hill plot, cooperativity.

## Results

### Occurrence of hemocyanin sequences in Myriapoda

**Fig. 1:**
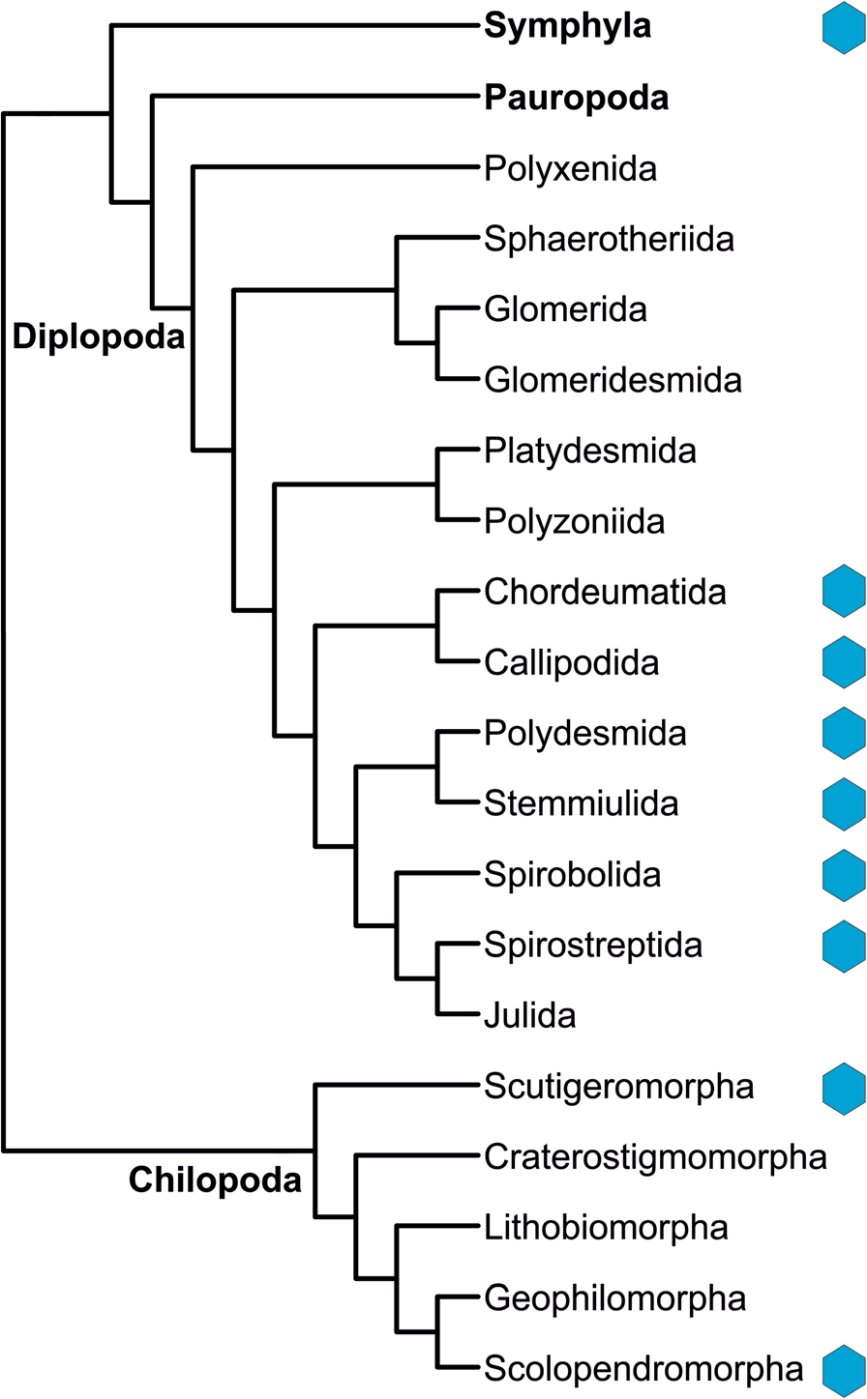
The occurrence of hemocyanin in myriapods. The phylogenetic interrelationships of the myriapod classes and orders were taken from [37]; the position of the Pauropoda was added according to a recent study [36]

We screened the available transcriptomes of 54 myriapod species [35–37] for the presence of Hc or PPO (Table 1). We found putative Hc sequences in the transcriptomes of 20 species belonging to Chilopoda (Scutigeromorpha and Scolopendromorpha), Diplopoda (Chordeumatida, Callipodida, Polydesmida, Stemmiulida, Spirobolida, and Spirostreptida) and Symphyla (Fig. 1). We included in the survey the transcriptomes of the venom glands of several scolopendromorphs and scutigeromorphs, which were found devoid of Hc mRNA. A Hc gene could also be assembled from the genome of *T. corallinus* (Spirobolida) [39]. By contrast, no *Hc* gene was found in the genome of the geophilomorph *S. maritima* [38]. Therefore, a total of 21 of the 56 (54 transcriptomes + two genomes) investigated myriapod species harbor at least one putative Hc genes.

The contigs that included *Hc* were extracted and the *Hc* cDNA sequences were deduced (Additional file 4: Data S1). In some cases, contigs were re-assembled or completed by back-mapping of the original Illumina reads. By this approach, we obtained 50 full-length *Hc* subunits sequences from the genomes and transcriptomes of 20 myriapod species. In the transcriptome of *Scolopocryptops sexspinosus* (Scolopendromorpha), only few *Hc* fragments were found, which could not be assembled. This species was ignored in our analyses. The sequences of the Hc subunits A – D of *S. coleoptrata* obtained before [32] were identical to that in the transcriptomes, except three isofunctional replacements in subunit B (ScoHcB). For ScoHcX, a highly divergent subunit that is not included in the native Hc [32], only a cDNA fragment was found, which displays multiple substitutions. In summary, this study revealed 46 novel Hc cDNA-sequences. The transcriptome of *S. dehaani* showed the presence of *Hc*-containing contigs, which could be assigned to two distinct subunits. The full-length sequences were obtained by 5′ and 3’ RACE methods.

**Fig. 2:**
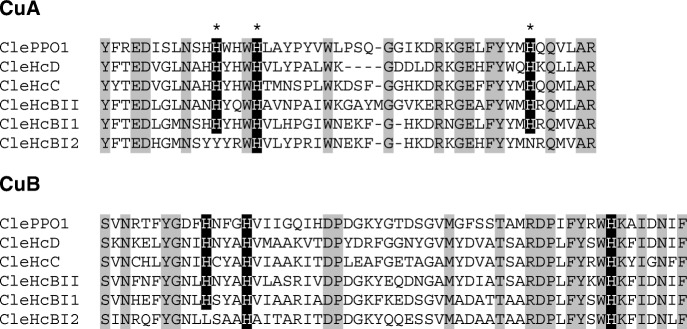
Copper-binding sites A and B of hemocyanin subunits and phenoloxidase of *Cleidogona* sp. Conserved residues are shaded in grey. The six copper-binding histidines are shaded in black and denoted by asterisks. Note the replacements of three histidines in CleHcBI2, which cannot bind copper

The cDNAs were translated *in silico* into proteins, which were included in an alignment that contained all other available myriapod Hc sequences [32–34, 56], resulting in a dataset of 57 putative myriapod Hc subunit sequences. These were assigned to specific subunit types on the basis of phylogenetic analyses (see below). We also added 16 PPO sequences identified in this or a previous study [34]. In all Hc subunits but HcBI2 (CleHcBI2) of *Cleidogona* sp. (Chordeumatida) the six histidines required for copper-binding and thus O_2_ transport are conserved (Additional file 3: Figure S1; Fig. 2). In both copper-binding sites (CuA and CuB) of CleHcBI2, one or two copper-coordinating histidines are replaced by another amino acid. Except for a low content of histidines (22 vs. 47 in CleHcBI1), no peculiarities in amino acid composition could be found in CleHcBI2.

### Molecular phylogeny of myriapod hemocyanin subunits

The multiple sequence alignment of the myriapod Hc and PPO sequences were subject to Bayesian phylogenetic reconstructions. As previous studies demonstrated the monophyly of the myriapod Hc subunits in respect to the Hc subunits of other arthropod subphyla [32, 34], we restricted the present phylogenetic analyses to the sequences of the Myriapoda. We employed a full alignment and, also, an alignment in which poorly aligned sections have been removed by Gblocks [47], and two different models of amino acid evolution (WAG and LG). The PPOs were considered as the outgroup.

**Fig. 3:**
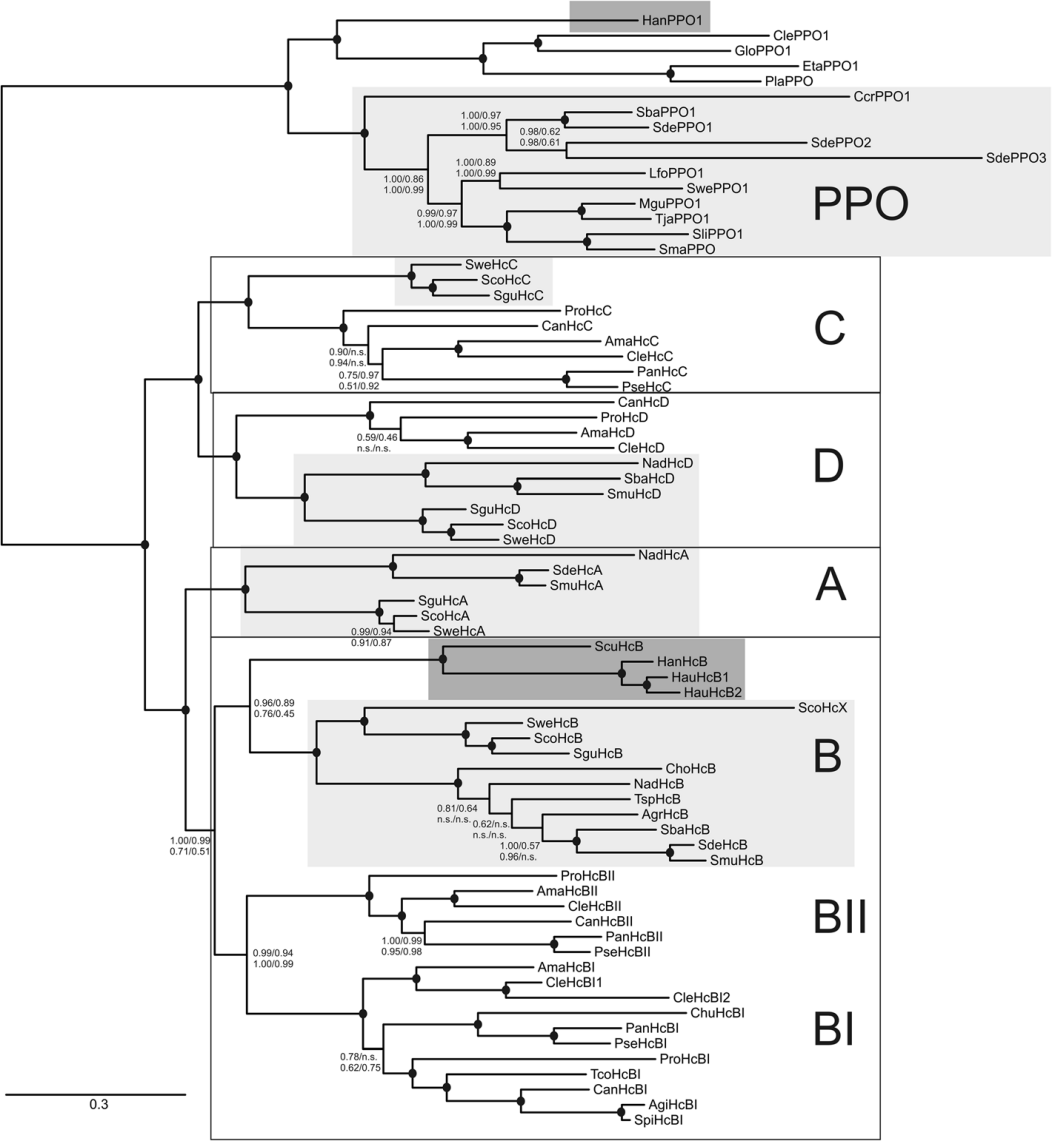
Phylogenetic analysis of the myriapod hemocyanin subunits. A Bayesian phylogenetic tree was inferred from an alignment of myriapod Hc and PPO amino acid sequences (Additional file 5: Figure S2). The displayed tree derived from the analysis of the full 868 amino acid (aa) alignment assuming the WAG model. The numbers at the nodes indicate the Bayesian support values according to the 868 aa alignment/WAG model, 531 aa alignment /WAG model, 868 aa alignment/LG model, 531 aa alignment/LG model. The black dots indicate support values > 0.98 in all four analyses. The bar represents 0.3 PAM distance. Chilopod Hc subunits and PPO are shaded in light grey; the symphylan proteins are shaded in dark grey; the different Hc subunit types are boxed and indicated on the right-hand side. See Additional file 2: Table S2 for the abbreviations

The four resulting trees were largely similar (Fig. 3). In all trees, we found five different types of subunits, which we named according to the subunit types of the centipede *S. coleoptrata* A-D [31, 32]. In phylogenetic analyses, A and B-type subunits on the one hand and C and D-type subunits on the other hand, form two distinct clades (Fig. 3). Within the C and D-type subunits, chilopod and diplopod Hc subunits are strictly separated. Symphylans only have B-type subunits, which associated with the chilopod B-type subunits, albeit with poor support (0.45 to 0.96 Bayesian posterior probability; BPP). In the Diplopoda, we did not find subunit type A, but two well-supported clades of distinct B-type subunits, which were thus named BI and BII. It should be noted that B-type subunits were found in all investigated myriapod species that have Hc. The copper-free Hc subunit HcBI2 of *Cleidogona* sp. is associated with the “typical” subunit HcBI1 of the same species.

### Estimating Hc subunit mRNA levels by RNA-seq

**Fig. 4:**
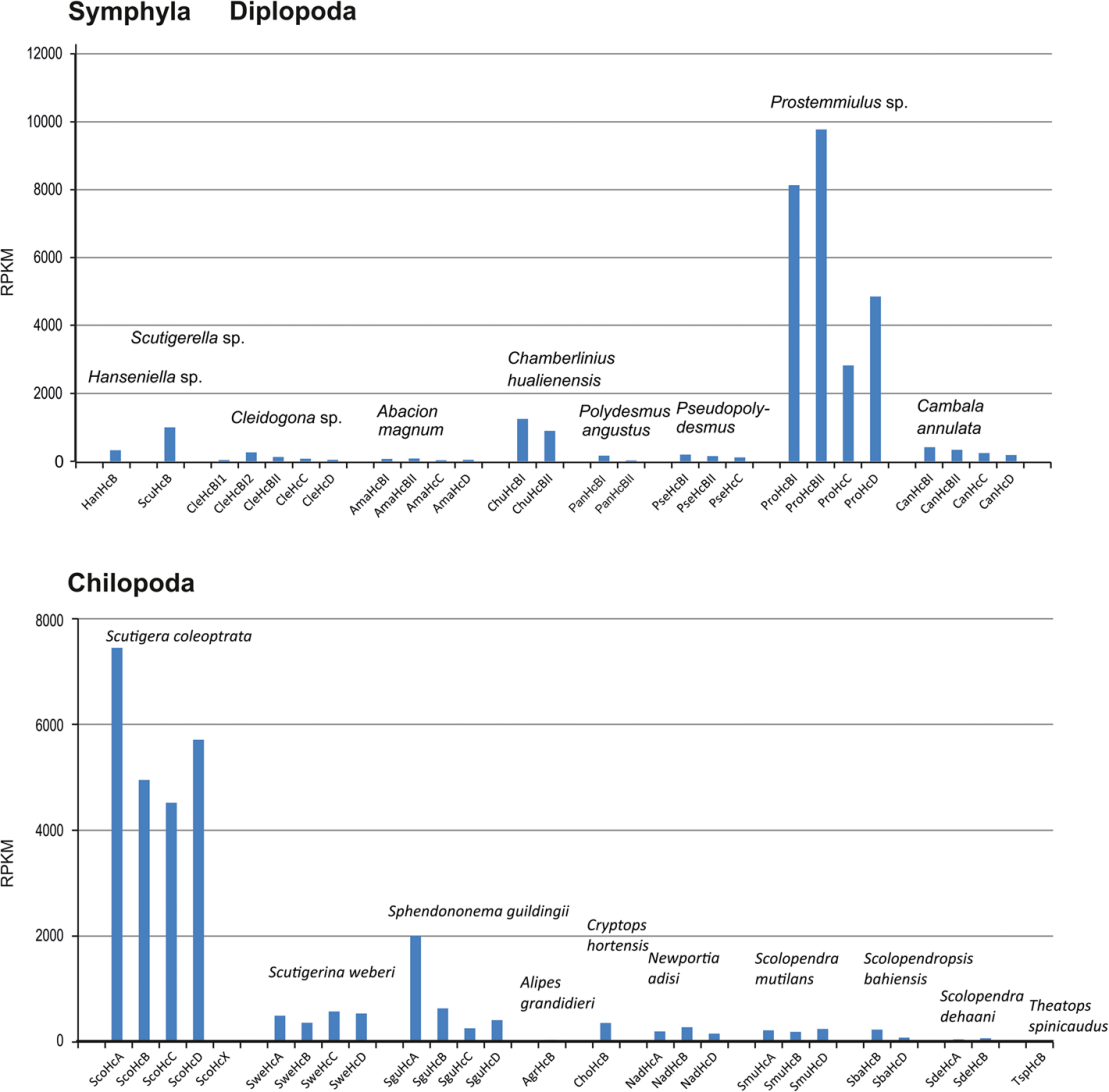
Hemocyanin expression in myriapods. The mRNA levels of Hc subunits were determined by RNA-Seq based on the transcriptomes (Table 1) and displayed as RPKM values. RPKM numbers are given Additional file 6: Table S3, the abbreviations of the subunits in Additional file 2: Table S2. A figure displaying the log-values is provided as Additional file 5: Figure S2

We studied the expression of Hc subunit mRNA by RNA-Seq, employing the publically available transcriptomes of 19 species (Table 1). The origin of the RNA used for RNA-Seq varied, although in most cases whole organisms or mixed body tissue have been used. We found highly divergent expression levels, which varied several orders of magnitude (Fig. 4; Additional file 5: Figure S2; Additional file 6: Table S3). The lowest Hc mRNA level was observed in the scolopendromorph *Theatops spinicaudus*, which has a single Hc subunit that displays 6 RPKM. *Prostemmiulus* sp. (Stemmiulida) had a cumulative RPKM of to 25,587.7. High Hc expression levels were also found in the Scutigeromorpha, with *S. coleoptrata* having the highest Hc expression levels (cumulative RPKM 22719.8). In most species, the RPKM of the different Hc subunits were in the same range, except the HcX-subunit of *S. coleoptrata*, which has a more than 1000-fold lower mRNA level than the other Hc subunits of this species. However, HcX is not a component of the native Hc and has an unknown function [32].

### O_2_ binding properties of *S. dehaani* hemocyanin

**Fig. 5:**
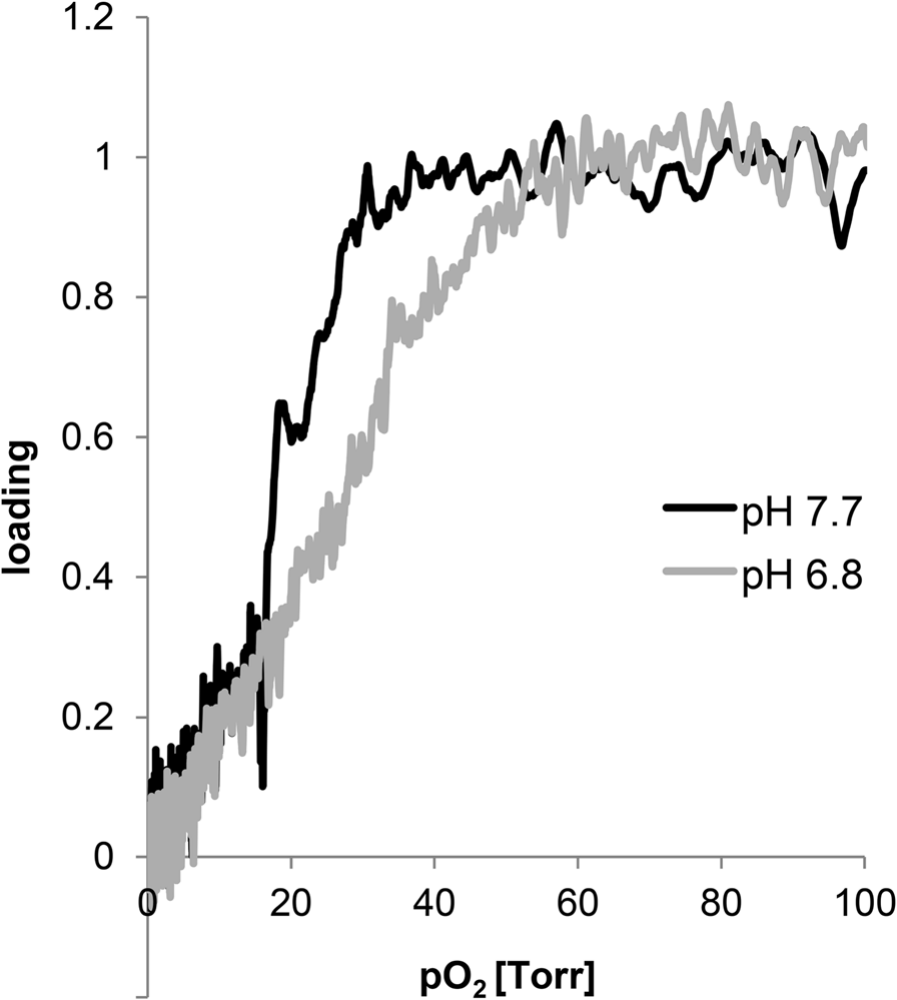
Oxygen-binding properties of the *S. dehaani* hemocyanin. Oxygen-binding curves were determined at pH 6.8 (grey) and 7.7 (black) by a polarographic-fluorometric method. Protein concentration was 0.2 mg/ml; the temperature was 20 °C

Equilibrium O_2_ binding curves of *S. dahaani* Hc were determined by measuring the O_2_-dependent fluorescence of the hemolymph of this species. Because there is no other respiratory protein in the hemolymph of this species and oxygen-dependent changes of the tryptophan fluorescence at this low oxygen concentration is specific for Hc, this approach is valid. We employed two different pH values in our measurements: At pH 7.7, the P_50_ was ~ 19 Torr (2.6 kPa), at pH 6.8 ~ 27 Torr (3.6 kPa) (Fig. 5). The reduced O_2_ affinity at low pH indicates that *S. dahaani* Hc displays the typical Bohr effect observed in hemocyanins. The sigmoidal O_2_-binding curve indicates cooperative O_2_-binding and a Hill coefficient (*h*) of 1.9 (pH 7.7) and 1.6 (pH 6.8) was calculated.

### mRNA and protein expression of *S. dehaani* hemocyanin

**Fig. 6:**
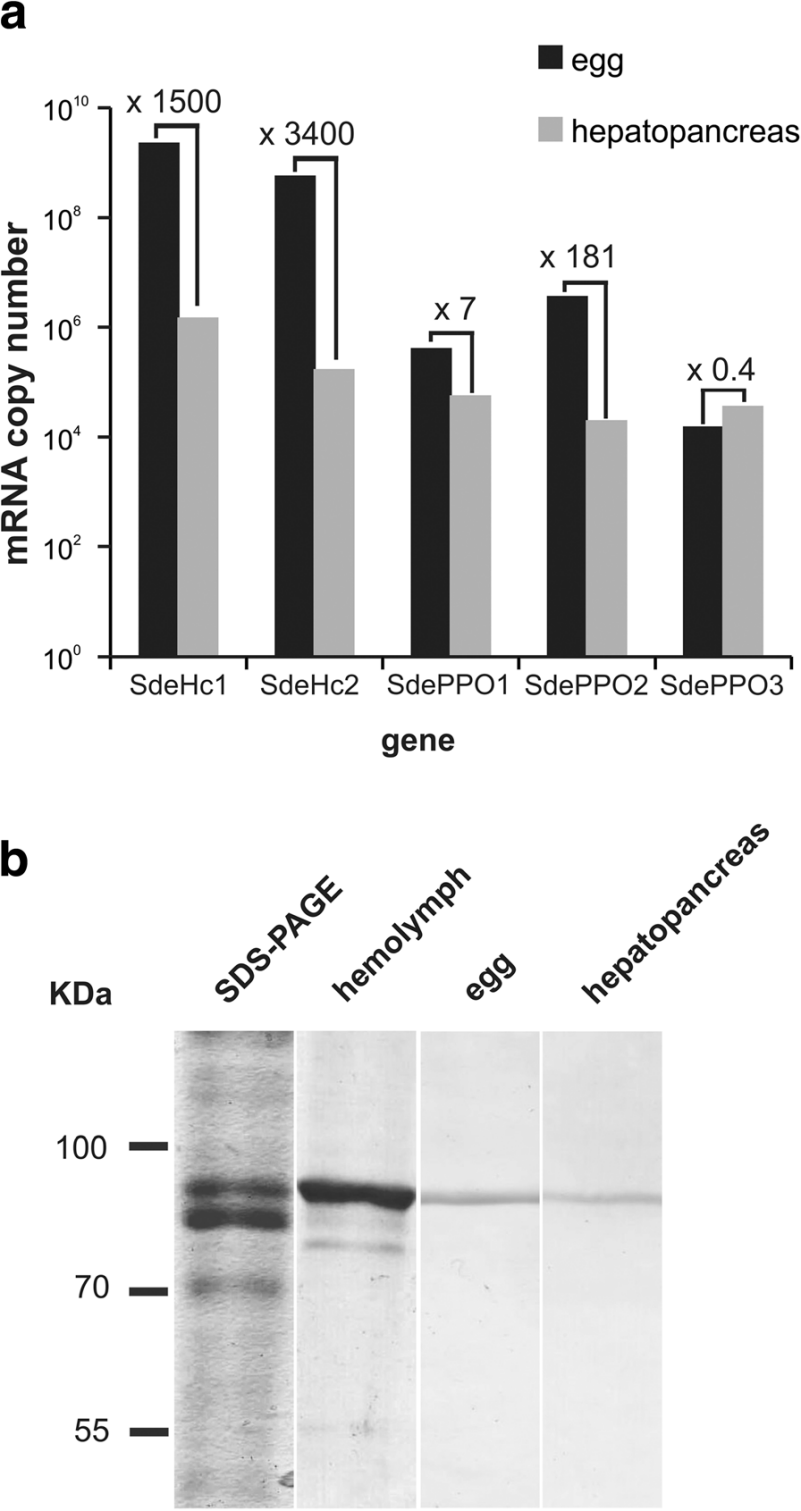
Quantification of the *S. dehaani* hemocyanin and phenoloxidase. **a** Levels of mRNA of Hc subunit and PPO in hepatopancreas and egg, as estimated by qRT-PCR. **b** About 5 μg total protein of hemolymph, egg and hepatopancreas of *S. dehaani* were separated by SDS-PAGE, and Hc proteins were detected using an antibody directed against *S. coleoptrata* Hc

Here, we investigated a female specimen of *S. dehaani* that carried fertilized eggs. Relative expression of Hc and PPO mRNA levels was determined using qRT-PCR in the hepatopancreas and the eggs. Notably, we found 3400-fold (SdeHcA) and 1500-fold (SdeHcB) higher mRNA levels in the oocyte compared to the hepatopancreas (Fig. 6a). The differences in the levels of PPOs were less pronounced, with factors ranging from 0.4 to 237. In addition, proteins were isolated from hemolymph, eggs and hepatopancreas, and analyzed by Western blotting. A single band with a mass of 80 kDa was detected (Fig. 6b). The relative band intensities were quantified with the software ImageJ, showing six-fold higher Hc protein levels in the egg than in the hepatopancreas.

## Discussion

For a long time, it has been assumed that – regardless of species and/or developmental stage – myriapods do not have Hc or any other respiratory protein. Only the Scutigeromorpha were considered as exceptions [3, 30]. However, more recent data have suggested that Hc is also present in other myriapods [2, 33, 34, 56]. A thorough survey on the occurrence, subunit diversity, and expression pattern was missing. Also, it was unknown why Hc was lost in some myriapod taxa, and whether Hc can significantly contribute to O_2_ supply.

### Widespread occurrence, losses and divergent expression levels of hemocyanins in Myriapoda

The examination of the available transcriptomes and genomes of the myriapods [35–39] showed that *Hc* genes are present in three of the four myriapod classes, i.e.*,* Chilopoda, Diplopoda, and Symphyla (Table 1; Fig. 1). No evidence for Hc was found in the transcriptome of Pauropoda. These findings confirm previous notions that Hc was present in the myriapod stem-lineage. Mapping of the occurrence of Hc onto the myriapod phylogenetic tree (Fig. 1) did not reveal a clear pattern. However, we must consider that a *Hc* gene may only be expressed in some developmental stages or certain tissues (see below), and might have been missed in this survey because the corresponding samples were not available. Thus, conclusive evidence for the absence of a *Hc* gene could only be derived from the genome of *S. maritima*. In those other species, in which no Hc mRNA was identified in the transcriptomes, at least a role of Hc in O_2_ supply in the adult can be excluded.

The phylogenetic pattern of the presence/absence suggests that multiple independent losses of the Hc gene occurred during myriapod evolution. Losses of *Hc* genes were also observed in certain taxa of Chelicerata, Crustacea and Hexapoda [5, 22, 28, 57]. Within Diplopoda, Hc appears to be restricted to the Eugnatha (except Julida), while Hc is absent in the early diverging diplopod orders. In the chilopods, Hc is present in Scutigeromorpha and Scolopendromorpha. While Scutigeromorpha split from the other Chilopoda early in evolution, Scolopendromorpha occupy a terminal position as the sister group to Geophilomorpha. Thus, there is no true correlation between the presence of Hc and myriapod phylogeny (Fig. 1). With the exception of Scutigeromorpha (see below), there is also no apparent morphological, physiological or ecological explanation for the presence of Hc, as the species with Hc have quite different body plans, behavior, and habitats. Thus, there must be others – yet unknown – features that have made a respiratory protein redundant in some species and explain its retention in others.

We also observed divergent expression rates across the myriapod taxa. Also in this context, we must consider that some variations in the RPKM may be due to differences in tissues or developmental stages used for transcriptome generations. There is no clear pattern across the taxa (Fig. 4), although in chilopods there is a tendency of high Hc mRNA levels in Scutigeromorpha and low Hc mRNA levels in Scolopendromorpha. The high mRNA Hc levels in Scutigeromorpha agrees with previous observations [3, 30]. A requirement of an effective O_2_ transporting system in the Scutigeromorpha can be explained by the high activity of this species, which is also reflected by the presence of a peculiar, highly branched circulatory system and tracheal lungs [3, 58]. The highest Hc mRNA levels were found in the diplopod *Prostemmiulus* sp. (Stemmiulida). To the best of our knowledge, there is at present no specific characteristics that could explain the high expression of Hc mRNA in this species. Notably, *S. coleoptrata* and *Prostemmiulus* sp. display approximately tenfold higher Hc mRNA levels (> 20,000 RPKM) than any other myriapod species. The very low Hc mRNA in several Scolopendromorpha (*S. dehaani*, *Alipes grandidieri* and *Theatops spinicaudus*) with RPKM < 100 raised the question whether Hc contributes to O_2_ transport, or whether this protein may have another function in those species (see below).

### Myriapod hemocyanin subunit diversity and evolution

Previous studies have demonstrated that distinct Hc subunit types occur in chelicerates, crustaceans, and hexapods [5, 10, 22, 28, 59]. Up to eight distinct subunit-types may occur in a single Hc molecule [25]. The presence of distinct subunit types is a common feature of arthropod Hc; they emerged independently in the different subphyla, and thus the subunit-types may have a long and independent evolutionary history of several hundred million years [10, 22, 28, 60, 61]. The presence of multiple distinct subunits has been associated with the controlled assembly to distinct quaternary structure, containing up to 48 subunits, and thus allowing establishment and regulation of high cooperativity in Hc or – if differentially expressed – may reflect specific needs during development and in response to environmental changes.

Most chelicerates have conserved 4 × 6-mer or 8 × 6-mer Hcs with seven distinct subunit types that emerged ~ 540 million years ago (MYA) [22, 25, 62]. The pattern in crustaceans is more complex and further complicated by the paraphyletic nature of crustacean and hexapod Hc subunits [60, 61, 63].

Myriapod species have between one and five distinct Hc subunits. Because the subunit HcX of *S. coleoptrata* is not present in the native Hc protein [32] and the copper-free subunit HcBI2 of *Cleidogona* sp. is also probably not part of the respiratory protein, the maximum number of distinct subunits in the native (oligo-)hexameric Hc is probably four. Notably, a B-type subunit was found in all myriapod species investigated here, suggesting that this polypeptide may be the central building block of the native Hc. The phylogenetic studies showed that orthologous C- and D-type subunits occur in both Diplopoda and Chilopoda. A-type subunits appear to be restricted to Chilopoda; the HcX subunit of *S. coleoptrata* is a B-type variant. In Diplopoda, two paralogous HcB subunits are present (HcBI and HcBII). Most likely, the duplication of the *HcB* gene was the response to the loss of the HcA subunit in this taxon. In a previous study [34], we identified two clades of Hc subunits in the myriapods, one which are built by the subunit types A and B and one which contains only C and D subunits. We could confirm this result in the current study. Further, the tree topology implies that the four distinct subunit types diverged before the Diplopoda, Symphyla and Chilopoda separated more than 500 MYA ago [35, 37, 64]. In case that multiple subunits occur within a single species, mRNA expression analyses showed that their RPKM values are similar within an order of magnitude. However, from the expression data, it is difficult to estimate the exact subunit composition of the (oligo-) hexameric Hc protein.

### A myriapod hemocyanin-like protein that does not bind O_2_

*Cleidogona* sp. (Diplopoda:Chordeumatida) harbors a phenoloxidase and five sequences that share significant similarities with the typical diplopod Hc subunits. Notably, we found that the HcBI subunit is duplicated. Closer investigation showed that in one of these subunits (which we called CleHcBI2) three of the six copper-binding histidines are replaced by another amino acid (CuA: H→Y, H→N; CuB: H→L; Fig. 2). Therefore, CleHcBI2 cannot bind copper and thus O_2_, and is unlikely to be integrated into Hc, or has a PO-like function. Phylogenetic analyses (Fig. 3) showed an about 4.5-fold faster evolutionary rate of CleHcBI2 compared to CleHcBI1, which is most likely due to the relief of the constraints imposed by the function of O_2_ transport. We speculate that CleHcBI2 carries out a similar function as the insect hexamerins or decapod pseudo-hemocyanins (cryptocyanins), Hc-related proteins which are used mainly for the storage of energy and amino acids [10, 16–18, 65]. Many hexamerins accumulate specific amino acids [18, 66], a feature that was not observed in CleHcBI2. The only notable difference in amino acid composition between CleHcBI2 and CleHcBI1 was the reduced relative amount of histidines (3.38% vs. 7.14%) beyond the replacement of the copper-binding sites. The phylogenetic tree (Fig. 3) suggests that this copper-free protein is not a common feature of myriapods or diplopods, but rather evolved specifically within the Chordeumatida. Nevertheless, it is remarkable that Hc has lost three times independently in different arthropod subphyla its respiratory function and probably evolved into a storage protein. This suggests that Hc has particular structural advantages, which may be for example their high stability or the ability to accumulate many amino acids with low osmotic impact due to their large size [17].

### Hemocyanin supports O_2_ supply even at low concentration

The low expression of Hc in many myriapod species raised the question whether this protein can in principle support O_2_ transport. We, therefore, analyzed the Hc of the centipede *S. dehaani*. This protein consists of two distinct subunits (SdeHcA and SdeHcB), and expression analyses showed RPKM values of 25.6 and 48.5, respectively (Fig. 4; Additional file 6: Table S3). We showed that the hemolymph and thus the Hc of *S. dehaani* does reversibly bind O_2_. Moreover, it displays a Bohr effect and cooperative O_2_-binding. These features strongly suggest a respiratory function of Hc in *S. dehaani*.

*S. dehaani* Hc displays a low O_2_ affinity (P_50_ = 19 Torr [2.6 kPa] to 27 Torr [3.6 kPa]; Fig. 5), which is higher than that of *S. coleoptrata* (P_50_ = 55 Torr [7.32 kPa] at pH 7.5) [3] but lower than the affinities measured for the diplopod Hcs (*Spirostreptus* Hc P_50_ = 4.7 Torr [0.63 kPa] at pH 7.5; *A. gigas* Hc (P_50_ = 3.45 Torr [0.46 kPa] at pH 8.1) [2, 33]. A similar pattern was found for the cooperativity, with high Hill coefficient for of *S. coleoptrata* (*h* = 8.9 at pH 7.5), an intermediate value for *S. dehaani* (*h* = 1.6 to 1.9) and low cooperativity for (*h* = 1.3 ± 0.2) for *Spirostreptus* Hc. These values show the flexibility of the O_2_ binding behavior of myriapod Hc, which most likely reflect differences in habitat and lifestyle. For example, a low O_2_ affinity and high cooperativity of *S. coleoptrata* Hc may be adaptive in efficient O_2_ release in this highly active species, whereas the high O_2_ affinity of *Spirostreptus* Hc may be interpreted as an adaptation for O_2_ storage function or efficient extraction of O_2_ in the sub-terrestrial environment. *S. dehaani* Hc appears to be better adapted to O_2_ release.

### The embryo may require high levels of Hc

We studied a female *S. dehaani* specimen with fertilized eggs. The exact developmental stage of the egg is unknown. Notably, we found much more Hc mRNA and also slightly enhanced Hc protein levels in the eggs than in the hepatopancreas, which is the principal site of Hc synthesis in adult crustaceans, hexapods and myriapods [27, 56, 67]. The relative Hc protein amount in the egg is probably underestimated due to the high concentration of yolk proteins. Our findings suggest that high levels of Hc mRNA are required for the early development of the *S. dehaani.* An embryo-specific role of Hc has also been demonstrated in the hexapods [68–70]. The accumulation of Hc mRNA in the fertilized egg may be required to meet the O_2_ requirements of the early development of the embryo and the restriction of O_2_ diffusion across the eggshell. In adult *S. dehaani*, the tracheal system additionally supports O_2_ supply, thus a lower concentration of Hc is required.

## Conclusions

Our results demonstrated that Hc was most likely the standard respiratory protein in the myriapod stem-lineage. Although Hc has been lost in certain taxa, it is still much more widespread in myriapods than initially appreciated. In previous studies, the presence of Hc in some myriapod species may have been overlooked due to the restriction to early developmental stages. Furthermore, some myriapods express Hc only at very low levels. Nevertheless, it is likely that Hc’s function is to support O_2_ supply by the hemolymph since the cooperative binding behavior is retained. Notably, in myriapods a non-respiratory protein evolved from Hc, which may act as a storage protein similar to insect hexamerins.

## Additional files

Additional file 1 **Table S1.** List of primer sequences used in this study. (PDF 64 kb)

Additional file 2 **Table S2.** List of sequences used in this study. (DOCX 24 kb)

Additional file 3: **Figure S1**. Multiple sequence alignment of myriapod hemocyanins and phenoloxidases in FASTA format. (PDF 101 kb)

Additional file 4: **Data S1**. Nucleotide sequences of the Hc and PPO cDNAs and gene identified in this study. (ZIP 81 kb)

Additional file 5: **Figure S2**. The mRNA levels of Hc subunits were determined by RNA-Seq based on the transcriptomes (Table 1) and displayed as log RPKM values. (TIF 3 mb)

Additional file 6: **Table S3**. Expression of hemocyanin subunit mRNA in myriapods. (PDF 72 kb)

## Abbreviations

Hc: Hemocyanin
MYA: Million years ago
P_50_: Half-saturation pressure
PPO: Prophenoloxidase
RPKM: Reads Per Kilobase exon model per Million reads; see Additional file 2: Table S2 for abbreviations of the hemocyanin subunits
