# Supplementary material for: Diversity, evolution, and function of myriapod hemocyanins
Source: BMC Evol Biol. 2018 Jul 5;18:107. doi: 10.1186/s12862-018-1221-2 (PMC6034248; doi:10.1186/s12862-018-1221-2)
Supplement: Supplementary file 1 — Table S1. List of primer sequences used in this study. (PDF 64 kb) [file 12862_2018_1221_MOESM1_ESM.pdf]

Additional Table A1: List of primer sequences used in this study

A) Gene-specific primers

SdeHcA For	GGACTGAACCTATGGCTGAAGGTTACG
SdeHcA Rev	CTGTCGGCCATTACCCCAACTTCTTCC
SdeHcB For	GTTGCTACATCTGCTCGTGATCCTC
SdeHcB Rev	CACTACGGGCTTGCCAGGTGTC
SdeHcX For	ATCCTCTTCTGAATCCAGTGCCAC
SdeHcX Rev	CTTCTTCGACATTCCCAACAAACCG

B) Oligonucleotide primers used for 5’ and 3’ RACE

5′ c19061 rev SdeHc1	TCAATGCCGTCCTCTTCACTAAG
5' N c19061 rev SdeHc1	CCAGTATGCGGTCTCTCCATTGT
V5′ lrc 10806 SdeHc2	AAAGATTCACTGCACTGGCATTCCG
V5′N lrc10806 SdeHc2	GAAGAGCTGAAAGTCAAGACCTTCGT
5′ c16444 rev SdePPO1	GGTCTCTCATAGTCATGTTAGCCG
5'N c16444 rev SdePPO1	CTTCAGCCATAGGTTCAGTCCAAT
5'lrc16015_r_3 SdePPO2	CAGCCGCATTGATGACTCGGTCGAA
5'Nlrc16015_r_3 SdePPO2	CACTCCACAGTAACTGGCGGCATCTA
5'c30121_r_3 SdePPO3	ACTTCTGCCACGAATTATCGCTAAT
5'Nc30121_r_3 SdePPO3	GTATTCCACAGTAACTTGGAGCAGT
3` c19061 for SdeHc1	CTATGAGGGAACGGAATTTCAAGTCT
3' N c19061for SdeHc1	GCTATTGTGGCATCCTCTTTGGTA
3` lrc10806 for SdeHc2	GACAAAGTCAAAGATAAAGTGGAACCG
3' N lrc10806 f SdeHc2	CGTCAATATTCGGTTTGTTGGGAATGT
3` c16444 for SdePPO1	GCCATTCCTGTTTCTGTTCGTTAC
3' N c16444 for SdePPO1	GACAACGCTTCTCTCCAAATGATC
3`lrc16015 for SdePPO2	GAAGATAGGGTGAAAGGGAAAGGTCA
3' N lrc16015 f SdePPO2	TGCGGCTGATTGGTCTTCATTCA
3' c30121 for SdePPO3	GTATCAACGCAGAGTCCATTACGGC
3`N c30121 for SdePPO3	TGTTGGTAATCGTAAATAGGGCTGTG

C) Oligonucleotide primers used for qRT-PCR

RTSdeHc1F	TCGCACCATTATCATTGGCACGT
RTSdeHc1R	GCATGTAAGCAAAGGCAGCACCA
RTSdeHc2F	TGCCTTGTCTGTGGCGATCC
RTSdeHc2R	TCCGGGAAAACATCCTGTATCCTC
RTSdePPO1F	TCAGGCCAGGACCATTCAGAGA
RTSdePPO1R	TCGCCAATAAGCCAGACGATGTT
RTSdePPO2F	TGGTGTTATGGCGGATACAGCAA
RTSdePPO2R	GTCAGTCTCGATTTGTGTTGCAGAAA
RTSdePPO3F	GGGCCACAATTCCCTTTCACG
RTSdePPO3R	TGGCAACATCTCCCATGACTCC
SdeActinF	GCTGCATCTTCAAGTTCCCTGGA
SdeActinR	GCCACAAGATTCCATGCCCAAGAA
SdeRPLP0F	TCGGTTCGAAACAGATGCAGC
SdeRPLP0R	GAGGGATCAATCTTTCCAATGCAGG
