# Supplementary material for: Diversity, evolution, and function of myriapod hemocyanins
Source: BMC Evol Biol. 2018 Jul 5;18:107. doi: 10.1186/s12862-018-1221-2 (PMC6034248; doi:10.1186/s12862-018-1221-2)
Supplement: Supplementary file 2 — Table S2. List of sequences used in this study. (DOCX 24 kb) [file 12862_2018_1221_MOESM2_ESM.docx]

**Table A2.** **List of sequences used in this study.** The accession numbers of the cDNA sequences or the next-gneration sequencing database sources are given. The cDNA and genome sequences derived from the transcriptomes and genomes are provided in Additional Data A1. Novel sequences obtained in this study are marked by an asterisk.

| **Abbreviation** | **Acc. No.** | **Protein** | **Species** | **Class** | **Order** |
| --- | --- | --- | --- | --- | --- |
| HanPPO1 | SRX1734405 | prophenoloxidase | *Hanseniella* sp. | Symphyla |  |
| PlaPPO* | HE574802 | prophenoloxidase | *Polyxenus lagurus* | Diplopoda | Polyxenida |
| EtaPPO1* | SRX1734393 | prophenoloxidase | *Eudigraphis taiwaniensis* | Diplopoda | Polyxenida |
| GloPPO1* | SRX326775 | prophenoloxidase | *Glomeridesmus sp.* | Diplopoda | Glomeridesmida |
| ClePPO1* | SRX326780 | prophenoloxidase | *Cleidogona sp.* | Diplopoda | Chordeumatida |
| SwePPO1* | SRX1637773 | prophenoloxidase | *Scutigerina weberi* | Chilopoda | Scutigeromorpha |
| CcrPPO1* | SRX1638413 | prophenoloxidase | *Craterostigmus crabilli* | Chilopoda | Craterostigmo-morpha |
| LfoPPO1* | SRX462145, SRX270896 | prophenoloxidase | *Lithobius forficatus* | Chilopoda | Lithobiomorpha |
| MguPPO1* | SRX1638910 | prophenoloxidase | *Mecistocepha-lus guildingii* | Chilopoda | Geophilomorpha |
| SmaPPO | genome | prophenoloxidase | *Strigamia maritima* | Chilopoda | Geophilomorpha |
| TjaPPO1* | SRX1638906 | prophenoloxidase | *Tygarrup javanicus* | Chilopoda | Geophilomorpha |
| SliPPO1* | SRX1638912 | prophenoloxidase | *Stenotaenia linearis* | Chilopoda | Geophilomorpha |
| SdePPO1* | LT841322 | prophenoloxidase | *Scolopendra dehaani* | Chilopoda | Scolopendromorpha |
| SdePPO2* | LT841323 | prophenoloxidase | *Scolopendra dehaani* | Chilopoda | Scolopendromorpha |
| SdePPO3* | LT841324 | prophenoloxidase | *Scolopendra dehaani* | Chilopoda | Scolopendromorpha |
| SbaPPO1* | SRX1734364 | prophenoloxidase | *Scolopendr-opsis bahiensis* | Chilopoda | Scolopendromorpha |
| HauHcB1 | HE574800 | hemocyanin SU B1 | *Hanseniella audax* | Symphyla |  |
| HauHcB2 | HE574801 | hemocyanin SU B2 | *Hanseniella audax* | Symphyla |  |
| HanHcB* | SRX1734405 | hemocyanin SU B | *Hanseniella sp.* | Symphyla |  |
| ScuHcB* | SRX1734406 | hemocyanin SU B | *Scutigerella sp.* | Symphyla |  |
| CleHcBI1* | SRX326780 | hemocyanin SU BI | *Cleidogona sp.* | Diplopoda | Chordeumatida |
| CleHcBI2* | SRX326780 | hemocyanin SU BI | *Cleidogona sp.* | Diplopoda | Chordeumatida |
| CleHcBII* | SRX326780 | hemocyanin SU BII | *Cleidogona sp.* | Diplopoda | Chordeumatida |
| CleHcC* | SRX326780 | hemocyanin SU C | *Cleidogona sp.* | Diplopoda | Chordeumatida |
| CleHcD* | SRX326780 | hemocyanin SU D | *Cleidogona sp.* | Diplopoda | Chordeumatida |
| AmaHcA* | SRX326781 | hemocyanin SU A | *Abacion magnum* | Diplopoda | Callipodida |
| AmaHcB* | SRX326781 | hemocyanin SU B | *Abacion magnum* | Diplopoda | Callipodida |
| AmaHcC* | SRX326781 | hemocyanin SU C | *Abacion magnum* | Diplopoda | Callipodida |
| AmaHcD* | SRX326781 | hemocyanin SU D | *Abacion magnum* | Diplopoda | Callipodida |
| ChuHcBI* | DRX028808 | hemocyanin SU BI | *Chamberlinius hualienensis* | Diplopoda | Polydesmida |
| PanHcBI | HE574797 | hemocyanin SU BI | *Polydesmus angustus* | Diplopoda | Polydesmida |
| PanHcBII* | SRX390267 | hemocyanin SU BII | *Polydesmus angustus* | Diplopoda | Polydesmida |
| PanHcC | HE574798 | hemocyanin SU C | *Polydesmus angustus* | Diplopoda | Polydesmida |
| PseHcBI* | SRX326779 | hemocyanin SU BI | *Pseudopoly-desmus sp.* | Diplopoda | Polydesmida |
| PseHcBII* | SRX326779 | hemocyanin SU BII | *Pseudopoly-desmus sp.* | Diplopoda | Polydesmida |
| PseHcC* | SRX326779 | hemocyanin SU C | *Pseudopoly-desmus sp.* | Diplopoda | Polydesmida |
| ProHcBI* | SRX326782 | hemocyanin SU BI | *Prostemmiulus sp.* | Diplopoda | Stemmiulida |
| ProHcBII* | SRX326782 | hemocyanin SU BII | *Prostemmiulus sp.* | Diplopoda | Stemmiulida |
| ProHcC* | SRX326782 | hemocyanin SU C | *Prostemmiulus sp.* | Diplopoda | Stemmiulida |
| ProHcD* | SRX326782 | hemocyanin SU D | *Prostemmiulus sp.* | Diplopoda | Stemmiulida |
| TcoHcA* | genome | hemocyanin SU A | *Trigoniulus corallinus* | Diplopoda | Spirobolida |
| CanHcBI* | SRX326783 | hemocyanin SU BI | *Cambala annulata* | Diplopoda | Spirostreptida |
| CanHcBII* | SRX326783 | hemocyanin SU BII | *Cambala annulata* | Diplopoda | Spirostreptida |
| CanHcC* | SRX326783 | hemocyanin SU C | *Cambala annulata* | Diplopoda | Spirostreptida |
| CanHcD* | SRX326783 | hemocyanin SU D | *Cambala annulata* | Diplopoda | Spirostreptida |
| SpiHcBI | HE574799 | hemocyanin SU BI | *Spirostreptus sp.* | Diplopoda | Spirostreptida |
| AgiHcBI | HE574797 | hemocyanin SU BI | *Archispiro-streptus gigas* | Diplopoda | Spirostreptida |
| ScoHcA | AJ344359 | hemocyanin SU A | *Scutigera coleoptrata* | Chilopoda | Scutigeromorpha |
| ScoHcB | AJ512793 | hemocyanin SU B | *Scutigera coleoptrata* | Chilopoda | Scutigeromorpha |
| ScoHcC | AJ431379 | hemocyanin SU C | *Scutigera coleoptrata* | Chilopoda | Scutigeromorpha |
| ScoHcD | AJ344360 | hemocyanin SU D | *Scutigera coleoptrata* | Chilopoda | Scutigeromorpha |
| ScoHcX | AJ431378 | hemocyanin SU X | *Scutigera coleoptrata* | Chilopoda | Scutigeromorpha |
| SweHcA* | SRX1637773 | hemocyanin SU A | *Scutigerina weberi* | Chilopoda | Scutigeromorpha |
| SweHcB* | SRX1637773 | hemocyanin SU B | *Scutigerina weberi* | Chilopoda | Scutigeromorpha |
| SweHcC* | SRX1637773 | hemocyanin SU C | *Scutigerina weberi* | Chilopoda | Scutigeromorpha |
| SweHcD* | SRX1637773 | hemocyanin SU D | *Scutigerina weberi* | Chilopoda | Scutigeromorpha |
| SguHcA* | SRX1637754 | hemocyanin SU A | *Sphendononema guildingii* | Chilopoda | Scutigeromorpha |
| SguHcB* | SRX1637754 | hemocyanin SU B | *Sphendononema guildingii* | Chilopoda | Scutigeromorpha |
| SguHcC* | SRX1637754 | hemocyanin SU C | *Sphendononema guildingii* | Chilopoda | Scutigeromorpha |
| SguHcD* | SRX1637754 | hemocyanin SU D | *Sphendononema guildingii* | Chilopoda | Scutigeromorpha |
| AgrHcB* | SRX205685 | hemocyanin SU B | *Alipes grandidieri* | Chilopoda | Scolopendromorpha |
| ChoHcB* | SRX457664 | hemocyanin SU B | *Cryptops hortensis* | Chilopoda | Scolopendromorpha |
| NadHcA* | SRX1638658 | hemocyanin SU A | *Newportia adisi* | Chilopoda | Scolopendromorpha |
| NadHcB* | SRX1638658 | hemocyanin SU B | *Newportia adisi* | Chilopoda | Scolopendromorpha |
| NadHcD* | SRX1638658 | hemocyanin SU D | *Newportia adisi* | Chilopoda | Scolopendromorpha |
| SmuHcA* | SRX286707, SRX286708 | hemocyanin SU A | *Scolopendra mutilans* | Chilopoda | Scolopendromorpha |
| SmuHcB* | SRX286707, SRX286708 | hemocyanin SU B | *Scolopendra mutilans* | Chilopoda | Scolopendromorpha |
| SmuHcD* | SRX286707, SRX286708 | hemocyanin SU D | *Scolopendra mutilans* | Chilopoda | Scolopendromorpha |
| SdeHcA* | LT841321 | hemocyanin SU A | *Scolopendra dehaani* | Chilopoda | Scolopendromorpha |
| SdeHcB* | LT841320 | hemocyanin SU B | *Scolopendra dehaani* | Chilopoda | Scolopendromorpha |
| SbaHcB* | SRX1734364 | hemocyanin SU B | *Scolopendr-opsis bahiensis* | Chilopoda | Scolopendromorpha |
| SbaHcD* | SRX1734364 | hemocyanin SU D | *Scolopendr-opsis bahiensis* | Chilopoda | Scolopendromorpha |
| TspHcB* | SRX1734363 | hemocyanin SU B | *Theatops spinicaudus* | Chilopoda | Scolopendromorpha |
