# Supplementary material for: Diversity, evolution, and function of myriapod hemocyanins
Source: BMC Evol Biol. 2018 Jul 5;18:107. doi: 10.1186/s12862-018-1221-2 (PMC6034248; doi:10.1186/s12862-018-1221-2)
Supplement: Supplementary file 3 — Figure S1. Multiple sequence alignment of myriapod hemocyanins and phenoloxidases in FASTA format. (PDF 101 kb) [file 12862_2018_1221_MOESM3_ESM.pdf]

>GloPPO1
M--A
YSFYSLLEKMEKPLGPESNT----RDPNGE-SFYPSNQER--QRILTE--IRRQGMDLNP
RGKPCSLFKWEVMEEALKTAIYITKI--SDLNEFLKCCAVLRDGINEEIYVYAFIYALLN
RTDTRGIRVPPIEEIMPYWYFPSEVITQASRKV-------KNAK-SKSERIVIPYT--FS
GNQLDPEFRVAYFREDLLVNSHHWHWHLVYPFTWLP-EM-----GGT-KDRKGELFYYMH
QQMLARYDAERLSNNLARVKPLSDIRSPIQEGYSSRLMSTLTGTFYPTRAANSKLSDVI-
-EISLARIQLWKDRLLEAIHTGEAL--GSDG---KKVKLIDP----------EGIDILGA
MVESAAISVNQKYYGILHNFGHVILSRIHDPDGRFGGDMGVMGFTQAAMRDPVFYRWHKE
IDNMFDEYKLNQKSYTTQDLT--WPDVVVSNVSVKAEKA---------------------
------------------------------QKANFLQTFWQEREVFLSRGINFVSEN-PV
VVSIRHLQHEDFSWNVEVDNTTGKEQEAMVRILIAPKYDEFQARYQFSDLRRIFIEMDKF
YAKLKPGKQTI-TRRSSSSNVTISTPQSFEVLANL--PPDA--PPKAEDAIC----GCGW
PDHMLLPKGRPEGMSFILFAMVSDFNKDKVQSS-------------RTT--LC--DDAAS
YCGILNQKYPDRRNMGFPFDRAAGTSVISLRDF--L--TPNMRTSTITIRHTGDVLGKDG
VVTPA---------TIF-----------
>ClePPO1
M--AAATNRQ
QGLLALFDHIGKPVVEDLSL----RGAGGV-SLIPSETQMIQIRTNLN--LRGQGIGAAP
RGKPCSLFKWENMQEAFALADLFTKL--GSYEEFVRTLAAVRPVVNEEICAFAQTYAFLH
RADTRNLSIPPTEEIMPHWYFPSTVITQADVAV-------KAAA-TEEERIVIPYE--FA
HNERDPEFRVAYFREDISLNSHHWHWHLAYPYVWLP-SQ-----GGI-KDRKGELFYYMH
QQVLARFDAERLCNSLNRVTPLNDFRKALDLGYFPNLISTNSGILYPGRPANMIPADLN-
-DISIDRMELWKDRILEAIHTRSVI--DRNN---KQTTLEDP----------AGIDILGA
IIESAALSVNRTFYGDFHNFGHVIIGQIHDPDGKYGTDSGVMGFSSTAMRDPIFYRWHKA
IDNIFNEYKLTLPNYTRQELT--WPKVILSHVSIKSKSS---------------------
------------------------------KKTNFIQTYWQEREILLSRNIDFGRAN-PV
VVRARHLQHESFVYELEVDNQTGGPQEGLVRLFLAPLLDELNDPLPLSELRRLFIELDKF
EVKLNAGRQVI-SRKSEDSNVTLTAARGFEQLATA--GEPT---PEGNDGFC----GCGW
PEHLLVPKGRPEGSRYTLFAMITDWSQDKVVSP-----------KPTTK--TC--RDAAS
YCGIRDQLFPDKRAMGFPWDRGVSRAA-TLDDF--L--TPSMSSREVTIRHSGEVVAVSG
VYQPT---------PVF-----------
>EtaPPO1
M---ATVTQKQY
THLITLLYQFSRPLGKQKLHPD-FHPEVDL-SFVPPDNELESYRQQAD--LPSRNIGQLQ
RRQPCVLFDQRIMTEATALADLLTEQ--KDFQDFLNVATAVRDVVNEELFVFALMSAMLR
RKETRDLPLPPIHEVIPYWYFPSEVFTRALREV-------RDSQ-DPQETIIVDYR--FA
GTEIDPEHKLAYFREDIDINSHHWHWHLVYPFTWAP-RD-----NEI-KDRKGELFYYMH
QQIVARYDAERLSNGLARVEPLSDFRYPIQEGYASQLTSISSGRHYSGRPANMVIRDTI-
-DISVQQMQMAKHRILEAIDNGYAI--NREG---QRVDLRDP----------KGSDLLGD
IVEAATISVNPRYYGDWHNRGHRIIMRIHDPDTRYGGDRGVMGFPSTAMRDPIFYRWHKE
IDNVFNTFKLTQPAYTDQELN--WADVTLTSISVQGVLS---------------------
------------------------------SSQNLLRTFWQEKVIPLSRGVEFAKPN-PV
LVQTRYLNHEPFLYNIAIHNRTDADIKATVRIFIAPQFSEFRREFTYAELRRLFMELDKF
QTTIAPGWQTV-TRQSSESSVTIPDATIFPWFDTTIPQEPENDGSGFRRTFC----GCGW
PDHMLLPKGRPEGMRFVLFAMLTDWNLDKVEGI-------------QEK--SC--CDPTS
YCGARDQLYPDKRSMGFPFDKPANTGVQTLDDF--MRNRTNMSKADLIIQHTGNIVPVGG
GIHEMG------EPEIR-----------
>PlaPPO
M---SKITQDQY
KALITLLYQFSRPLDKQKQHP---YPEVDL-SFVPPENELEEYRQRID--LEGHHVGQLP
RRQPCELFDPQVMNEATTLAQIFTEQ--QNFDEFLRVATAVRDVVNEELFVFALLSAILK
RPDTTDLPLPPIQEIIPYWYFPSEVFTRALRRV-------RDAQ-NDQEEIVLDHP--FA
GNELDPERRLAYFREDVDVNSHHWHWHLVYPFTWVP-QQ-----GEV-KDRKGELFYYMH
QQIMTRYDAERLSNYMARVQPLSDLRAPIREGYASQLTSIVSGRHYSGRPAHMVLSDTI-

```
-DVSLQRIEQAKSRIQEAIHRGYAI--DRQA---KQVPLRDP----------KGIDTLGD
MVEATVLSVHREYYGDWHNHGHRIISRIHDPDTRYGGDRGVMAFTGTAMRDPIFYRWHKE
IDDVFNEYKLTQTPYTNEELI--WNQVKVTGVSVAGEIS---------------------
------------------------------TSPNLLRTFWQEKRVPLSRGVDFGRPN-PV
YVLTRHLQHEPFLYNIAVHNSTDKEVTATVRLFMAPKFSEFRREFTYTEIRRLFFELDKF
QAKIAPGWQTV-TRLSDESNVTIPDYTNFTWFETN--PELGTRPGGFRETFC----GCGW
PNHMLLPKGKPEGMKFILFAMLTDWNQDKVNGA-------------TKQ--KC--CDPTS
YCGAKNQLYPDKKSMGFPFDRPASAATRSLGDF--LRDKSNMLTTDVVLVHTGQIIPLGG
DITSAR------DPEIF-----------
>MguPPO1
M----------------------------------------------------TTTRTMQ
RAVLDLFEHVTEPLDRRPAF----QASIS--GVLPDPKDLAETRDRLG--PLLRGIGILP
RGRIFSGFRRTHMEEAMRVIKLLMGF--NDNRDLQLAMTYLRDFINEEMFVYVTSVLVLH
KPQLRGINLPPIEEVLPQKFVPSDIVSRAVNQE-------RRTPAESTEPIILNVD--FT
GNDLDIEHRLAYWREDVGLNTHHWHWHIVYPFTWEA-SL-----GGM-KDRKGELFYYMH
QQMIARYDCERLSHNLSRVVSYHNFDEPIPEGYAPHLSKDISGTVYASRPANMIMRDLP-
-SITVNRMRIWYNRIIDAIHRGNYI--QSDR---TRVPFDAA----------TGIDKLGD
ILEAATISPNITYYGDFHNEGHNLLSRIHDPDERYREEMGAIGDPATAMRDPIFFRYHKF
IDLIFQEYKNTLPPYTPEQLR--WDDVAVQTITLQGTVS---------------------
------------------------------QRPNNLQTFWEDRQFDLQRGINFNRPT-PV
SIRTRHLQHETFSYSIVLQNSASVARTGTVRIFMAPTRNEINQPFIADVQRHLFIELDKF
EATLNPGANTV-TRQSSESTVTLSGLKSFGQLIQE--SQSEETPNEEQVSAC----TCGL
PDHLLIPRGKPEGMEFQVFVMVTNAEEDRVNEP-------------QTQ--RC--RDAPS
YCGVLNERYPDRKAMGFPFDRRIVFG--SWGDF--Q--TPNMGFTTVTVRFRNEVVDTSG
RSVLF---------PIN-----------
>TjaPPO1
M----------------------------------------------------TDRQLLQ
QTVLDLFEHVTEPVDRRPPF----QASIP--GIVPDAKDLADTGVRLG--PLMTGIGILP
RGRIFSGFRRSHMEEAMLVVKLLMGM--NETRDLRLAMTYLRDVINEEMFVYATSVLVLH
KPQLQGVNLPPIEEVLPQKFVPSEIVTQAVNEE-------RRTPAETTDPIILSVD--FT
GNDLDIEHRLAYWREDVGLNTHHWHWHIVYPFTWEP-SL-----GGM-KDRKGELFYYMH
QQMIARYDCERLSHNLSRVTSFHNFTEPIQEGYAPHLSKDISGTVYASRPRNMVLHDLP-
-SMTVQRLRTWYNRIIDSIHHGSYL--QPDG---TRVPFEEV----------TGIDRLGD
IVEAAVISPNFTYYGDFHNEGHNLISRIHDPDERFREEIGVMGDPATAMRDPVFFRWHKF
IDLIFQELKNTLTPYTREQLT--FADVSVQTITVQGTLS---------------------
------------------------------QRPNNLQTFWQERQFELQRGINFNRPT-PV
SIRTRHLQHEVFSYSIVAQNAGSDARTGTVRIFMAPARNEISQPFVPNDQRHLFIELDKF
EATLNPGVNTI-ARQSSVSSVTLNDIKSFNTLIQE--AQT-ETPSPEEVSAC----TCGL
PDHLLIPRGKPEGMEFQIFVMITNAEQDRVASG-------------ATQPAQC--RDAAS
YCGVLNERFPDSKAMGYPFDRRITAG--AWVDF--Q--TPNMGFSTVTVRFRNEVVNTTG
SSVLF---------PID-----------
>SliPPO1
M-----------------------------------------------------SDVDLQ
RVVLDLWDHVSEPLDRRADF----QRPFAE-GAVLDNRDLAETGERLR--PRLTGIGVLP
RGRIFSAFNKMHMAEAMRVVRLLQSM--TDDRDLALAMGYLRDHINEQMFVYVTSVLAVH
RPSMRNVQLPPIEEVLPSKFIPAPLITQALNDE-------RRQQPAAAEPIQLSLD--FT
GNDHDIEHRLAYWREDIALNAHHWHWHIVYPYTWTS-EM-----GGT-KDRKGELFYYMH
QQMLARYDCERMCHHLSRVESYDNWEDPITVGYAPHLNNDISGTEYASRPLNMVLQDIP-
-AVPVHRLKTWYNRIINSIHEGFYR--QTDG---KLLPFDEA----------TGVDKLGD
MVEPAAISPNPIYYGEFHNSGHDLISRIHDPDERFREEIAVMGDPATAMRDPVFYQWHKF
IDLIFQEHKNTLTSYTTQQLQ--WNDVNVQSAEVKG-MS---------------------
------------------------------SRPNILFTFWQTRTIELQRGINFNRST-PV
RIQYQHLQHEAFNYNIVIENTTSNPVEGMIRIYMAPSSNEIQQPFQLEQQRHLFIELDKF
ISILQPGSNTV-TRRSSESNVTVATQKNFSQLVQE--SQDGSTPPTEQLSYC----SCGL
PDHLLIPRGKPEGLEFQLFVMVTNGREDRIVST-------------TPN--RCGADSATS
```

```
YCGVLDDKYPDRRPMGFPFDRRVEAA--TLDAF--R--LSNMSITPVIIRFRNVVMGTND
GSILF---------PSE----------T
>SmaPPO
M-----------------------------------------------------TTMNPQ
RVILDLWDHVSEPLDRRATF----QRPFEG-APVLDNRDTAEIGGRLQ--SRLVGIGILP
RGRIFSTFNKQHMAEAMLVIRLLQDLVTSDLRDSLLAMGFLRDRINEQMFVYVTSVLTLH
QPELRNIRLPPIEEVLPMKFVPGPLITDARNTE-------RRMPADATEPIVLSLD--FT
GNDRDIEHRLAYWREDIALNTHHWHWHIVYPFTWTP-DL-----GGT-KDRKGELFYYMH
QQMIARYDCERMSHNLSRVVVYHNFDDPIDEGYAPHLNNDISGTVYASRPRNMVLQDTP-
-SVPVHRLITWYNRIVNSIHERTYR--RSDG---QVVPLDET----------NGISIIGD
IVEPAATGPSPTYYGDFHNSGHDLISRIHDPDERFREEMGVMGDSATAMRDPVFYRWHKF
IDQIFQEHKNTLDPYTPEQLQ--WNDVTVQSVSVQGSTS---------------------
------------------------------SRPNNLQTFWQDRTFELQRGINFNRSI-PV
RLQTRHLQHEAFTYNIVIQNGSSSAVEGMIRLFMAPSNNEIQQPFTLEDQRHIFIELDKF
TATLNPGNNTV-TRQSSQSNVTVATQKSFSQLIQE--SQDGT--TEEQISSC----SCGL
PDHLLIPRGKPEGMEFQLFVMVTNAQQDRVVPS-------------TTT--TC--QDAAS
YCGILNERYPDVRPMGFPFDRRIEAA--TLDVF--R--TTNMNMAPVNIRFRNVVVGTND
SSVLF---------PNE----------R
>LfoPPO1
M-----------------------------------------------------ARPGMQ
RVMLDLFEHVTEPLESRAAF----QNAFSG-GPVINSRDAAEVEERLK--NQMGGIGQLS
RGRMFSVFRKQHLNEAMRVVKLLMGL---EDRDLQLAMAFLRDRINEELFVYAASIVTFH
KPEMRMVRLPPIQEILPEKFFPADIISRARNME-------RRQS-EPQTPLVIGLD--FT
GNDLDIEHRLAYWREDINLNAHHWHWHIVYPITWIP-EM-----GGV-KDRKGELFYYMH
QQMIARYDAERLSHNLRRVESFDNWSDPMQEGYAPHLSKETSGTVYASRPVNMVLKDLP-
-DVTITRLQTWYERILDSIHRGFIL--LPDG---TRERLDEI----------TGIDKIGD
IVEASLLSPNPDYYGDFHNNGHDVISRVHDPDGRFREEMGVMGDTATAMRDPIFYRWHKF
IDLLYQEYKRTLLPYS-THLI--WNDINIEEIKVQGRLS---------------------
------------------------------QQVNALQTFWEDSEIRLDRGLNFNSPT-AV
SVMVRHLQHEAFQYTLDIENRTSTPHSGTVRIFLAPRLNELQEPFPLNDQRHLFMEMDKF
EVSLKPGKNII-HRNSADSNVTLSNIKTFSQLIEE--VTES---SEEQQSYC----GCGL
PDHLLLPKGKPEGMSFQLFVMVTNSNEDRVTGG-------------SGQ--KC--QDAAS
YCGILNEKYPDSKPMGFPFDRNIEAA--NLADF--K--TSNMSFTDVIIRFRNEVLDTNG
STVFF---------PGA-----------
>SwePPO1
M-----------------------------------------------------SASPPWQ
RLVLDLFDHVDEPFDTRRLT----PRPWSNEGPLIAARDRASAGTTIQ--PRLRSIKRLQ
RGKMFSIFHRPHMEEARGIIQLLQGM--SDTRELQSALSYLRDDINEEMFVYIVSVLLYN
RTDSRGITFPPIEEVLPAKFFPSEILSRARNED-------RRLT-DTTTPITIDMD--FT
GNDLDIEHRLAYWREDIGLNAHHWHWHVVYPFFWTP-EM-----GGL-KDRKGELFYYMH
QQMLARYDTERLCHNLSRVETLHNWVDPIPEGYAPHLSKEISGTIYASRPANMTLQDIP-
-QIQLHRLRTFYNRIIESIHVGSIL--LPNG---QRQPLTEQ----------TGIDVLGA
IVEPSSQSPHPNYYGDFHNNGHDVIARIHDPDGRFNEEMGVMGDTSTAMRDPVFYRWHRF
IDDLYQEHKRTLNPYTRQQLT--WSAVAVRSVSVQGKIS---------------------
------------------------------QSSNVLQTFWQDREIFLQRGMNFNSPR-PV
SIRVRHLQHETFSYTIAVENTTNRPVEGTVRIFLAPVQNEIREQFPVDEQRLLFMEMDKF
AISMKPGANTL-QRVSTQSNVTVATVRSFSDLIND--ANQPG--AGEAVSSC----SCGL
PDHLLLPRGKPEGMAFQLFVMITNAAEDKVEGS-------------SGR--RC--MDAAS
YCGVLNEKYPDRKPMGYPFDRRIQAP--DIETF--L--TPNMGIQEITIRFRNIVSDPTG
TRVLM---------PDT-----------
>SdePPO1
M-----------------------------------------------------AEVKSMQ
RLVLDLFDNVSEPLDQRQPF----HTAFAS-GPVITSRDAAATGTRLR--PQMTGIGILP
RGRIFSTFNRDHMDEAMRVVRLLMRL---DDRDLQLALAYLRDNINEELFVYSISVLSTS
QPTLRRTKLPPVEEVLPAKFIPSSAISQARTIQ-------RRASQETQETIIIDVD--FT
```

```
GNDLDPEHRLAYWREDIGLNAHHWHWHIVYPYTWIP-EL-----GTI-RDRKGELFYYMH
QQIIARYDCERISLNFPRVRPLLNWTEPMAEGYAPHLSKEISGTVYAFRPANMTMRDLP-
-FITVDRLRTWYQRIIQDIHKGSFT--RDDG---TKVQLDND----------QGIDIVGD
VVEAAYTTPNATFYGDFHNLGHQIIARVHDPDERYKEEVGVMADSTTAMRDPIFYRWHKF
IDNVFVEHKMKLKPYTEEELT--FKDIVVNGIQVQGKMS---------------------
------------------------------QRPHIIGTFWQERTISLQRGITFNSAI-PV
SVRYRHLQHESFTWNIEVENKSNADKPGTVRIFMAPTFNDTRQRFSPNDQRVLMLEMDKF
EFNFKPGKNII-QQRSEQSNITIPTLRSFGELIAE--SESDD--LLDAQSYC----SCGL
PEHLLVPRGKQEGMPFQTFVMITDAVKDRVNGD--------------SK--QC--HDATS
YCGVINEKYPDKKPMGYPFDRVILAA--DWSTF--K--TSNMNFGDVTVTFKEEVINANA
GSLVF---------PK------------
>SbaPPO1
------------------------------------------------------------
-----LFDNVHEPLEERKSF----QAAFAS-GPIIRSRDAAATGERLR--PQMNGIGILP
RGKIFSTFSKDHMDEAMRVIRLLMRL---ESRDLQLALAYLRDNVNEELFVYATSVLFTH
QPNFRGIRLPPVEEVLPAKFIPSDVISRARVIQ-------RRASTESTEPIIIDVD--FT
GNDLDPEHRLAYWREDIGLNAHHWHWHIVYPWTWTP-DM-----GTI-KDRKGELFYYLH
QQMIARYDCERMSLNFSRVTPLQNWNEPMAEGYAPHLTKEISGTVYAFRPANMRLRDMP-
-HITVDRLRTWYNRIVQDIHKGAFT--ASDK---KKVPLDND----------KGIDIVGD
VVEATYNGVNPTFYGDLHNLGHWTIARVHDPDERYKEEVGAMADSTTAMRDPIFYRWHKF
IDNLFHEHKFTLTPYIKEQLT--FADIVVNAVRVQGKVS---------------------
------------------------------QRPNMIGTFWQDRTLNLERGVAFNSPV-PV
AVRIRHLQHESFTWNIEVENKSQQDKQGTLRVFMAPTFNDTRQRFSPNDQRLLMMEMDKF
EFTLKPGKNTI-QRRSEDSNVTVPSLRSFGQLIAD--SEGGNQ-SQEAQSFC----SCGM
PDHLLVPRGKAEGMPFQTFVMITNALEDRVEGG--------------TE--KC--RDATS
YCGVLNELYPDKKPMGFPFDRVIHET--DWSLF--K--TPNMAFGDVSIQFREQVVNASA
GNLMF---------PE------------
>SdePPO2
M----------------------------------------------------EDQLNLQ
KLVLDLFRNVSEPLSESDFPV--FATSYAG------------NKDIFY--EFEEKIGILP
KGRIFSTFKREHMKEAIDIIELLTKT---ETNNLDPIMLKLRKKVNEELFVYATSVFFIQ
NPDLK-IKLPLIEEVLPQKFCTGDVIRKAVKEN-------E--KAMSMIPIIIDVE--YT
STPLEPEHALAYWREDIGLNAHHWHWHIVYPYEW-P-DG-----NKP-KDRRGELFYYMH
QQMIARYDCERLAVDLPRVVPLHDWDNFIPEGYAPHLNKEIANTIYCYRPSNMILN----
-QVNIDELRQMYETIIQDLRSGFFI--RNDD---AKIPLDTE----------RGIDIVGN
TVEASNASANMDRYGSLHNNGHNAIARIHDPDGRFKLEDGVMADTATAMRDPIFYRWHKF
IDNIFLQHKSRLTPYTREELM--FEKVIIDSIYIQGKTS---------------------
------------------------------KATDKIETFWQESKLNLENGINFKNKV-SV
SVRYRHLQHEKFEWNIEVENKWNENEQGTVRIFMAPIYGEDGKELSPNEQRLLMIEMDKF
EYTLRPGTNYI-SRRSDQSNVTIQTLKRFSELDKE--TK------VTNEASC----SCGL
PDHLLVPRGKKDGMAFQTFVMITSAIEDRVKG--------------KGQ--HC--LDAAS
YCGVINDKYPDKKPMGFPFDRVINAA--DWSSF--K--TPNMGFGVIKICFEDIVKDSDL
HDE-----------NDH-----------
>HanPPO1
M-------------------------------------------------------EEGY
RRILTMLRHSATDITYTF------PAPLSG------------AGVIDRVIPAIPNLGRLQ
RGRSFSTFNRSHMQEAMQIVRAFTGL--ENLDECTDLASTLRSRINEELFVYCMYSTVLH
RPDTRNAMMPPIEEILPYKFVDNRVIKRAWRTL-------ESRQPDQLEPIEIPYN--FS
GNDLDPEHRLAYFREDIGINSHHWHWHIVFPIEWRA-SD-----GGL-KDRKGELFYYMH
QQIMARYDAERFSLHMARTRPLQNWTLPVPEGYSPHLTSELSGQEYANRPSFMTMEDVPG
DSITLDRLQTFRNRIIHAIHTGTII--DSNG---ETRRLDEQ----------TGIDVLGD
MIEASSLSPNMQFYGDFHNLGHIVLARMHDPDRRFLETNGVMGFSETAIRDPVFFRWHRF
IDDIFVDYKETLTPYTTEQLN--WSGVSIQGVTIQGNGT---------------------
----------------------------NAGTANQLETFWQERVLTLSVGVNFTRDV-PV
TVKLTHLQHEEYDLVLDVDNSADSVVTATVRVFLAPTLDEASARLPLNQMRRLFIELDKF
```

QTTVEPGRQTI-QRRSMDSNVTIPSPQSFGDLLNL--ALP----STVEQSSC----SCGW
PHHMLLPRGRPEGMNFTLFVMVTNGIEDQVEQV------------SSER--RC--ADATS
YCGIRDERYPDRKPMGFPFDRRFEQE--LLSSI--Q--TPNMVTADVVIVHSGRILDVSG
NSRLVRLEDLGGTVPSTGTTPLEGPVWP
>SdePPO3
M--EKTRQMQ
GFILDMFNKVSESHPRHKHE----HILASH-GIQFSSY----FETETF--LKSEGLGVLK
KGKIFSPFNNEHMDEAFKAIKYLNDT-KLDTKSLIIKIVGLHMLLNEELFVYVTSYVIIN
HPDFKTVHLPPYQEIFPDKFIHKETISKIKRQV-------IHNGKDSKKEIVIPAD--FS
GDNLDPEHRLAYWREDIQLNSHHWNWHLVYPTDWTP-DS----GEGL-RDRKGELFYYMH
QQLVARYNCERLSVHYPRVTAFFEWDEPIVEGYNSHLLKENSGINYGPRPGNMILQDLP-
-NLTKSILRAWYQRIMQSIHLGYLT--SREYN--THIPLNNE----------GGIDKIGA
IVEASRLSVNYDFYGNLHNMSHNSLSRIHDPDERYKGDPGVMGDVATTVRDPIFFRWHKF
IDDIFQEHKLQLPSYNEEGLM--FKDIIVKSVDTEG------------------------
------------------------------KQNNQLQTFWEESTLDMKYGLAFECGS-PI
VVRYRHLQHEQFNWSIIVENISNEKKQGMVRIFMAPKYDVTGNQFTPNELRKLMIEMDKF
EFTFEAKKTVIVTRKSRDSSVIIPDAKTTQNIREE--LQK----DDPWQHYC----GCGL
PSHLLVPRGKREGMNFMTFVMITDATKDKVYEE-------------DDD--VC--KTAPS
YCGILNKKYPDKRAMGFPFDRQISDN--SWQKF--Q--KPNMSVENLNSDLLVIVNRAVK
NSL-----------AIL-----------
>CcrPPO1
M-NL------AKLTSVCVLGLSV--------------------FLL-C-GS-AVAHKGIQ
KHITGYLYRLTVPSDTDGPLSNSSGPPFAG-SSTNSTTEFVDIEVRYPDLNTLCGPGKLP
RGRVFSAYQRDHMRQVEDITIFIMKI--NNETEVIEVLGFLRNKVNEQLFLYVVSVLVLV
HPQMAGINLPLIQDVLPNKFITCEMISEVRQDILVNVSGTKERQ-AEPQVMVLDQD--FT
GSNINPEHRVAYWREDIGLSAHHWHWHVVYFSDFLPISA-----DQT-RDRKGELFYFMH
AQLLARHNSERLASRLARIMPLKDWSQRIPEGYDSHLGNEISGVVYAPRPADMLMKDVPG
-QIQLNEMQTWYDHIVDTIDRQQFL--RRSGNNFQTVSLNDE----------SGIDTLGN
MLESNAMSPDLTYYGDLHNMGHVVLSRIQDPTGAFKTEMGAMGDLVTAMRDPVFYRWHLF
IDDLYVRHKRTLPTYSDRMFN--FQDVDISSVVVTGDVT---------------------
------------------------------KTPNKLVTYWQSEQLNLAHGLDFRRRN-SV
TVRFQHLQHENFKYTITVNNKLVTNTLGMVRIFWVPTLNEAGEPFDLEDQRRFFIEMDRF
PATFTPGNNVI-TRLSSESAVILPSRASFAQPNSF--DEL----GSIGNSYC----TCGL
AEHLLVPRGTEAGMQFQMFVMISNGTADRVVDLP----------PDSKA--SC--KAGVT
LCGILNRKYPDSKPMAFPFDRPFRGGQSSISEF--AQGRSNMFLTDMTGTGSG-------
---------------------------F
>HauHcB2
M-RS-------LIVFCGLLALAIA-------------------AEN-C-GE-KTDVEAKQ
AKVKEVIQYINRPHIHSGG-----SCD------------AE-ASHLTA--ASLKEIGHLD
TNSVFSLFDERTWPEAKEAVRLMMKA--ANFDEFITIATDLSHHMNDDMFLFCFNVAIVH
RDDTSGVRPEFHFNSFPDKYIRHDQIVEIRHAV-------ECGQ---DNIKVLANP--HV
FNDTDDAHKLLYWLEDLGLNAHHYHWHTVHPAIWTE-EL-----GRG-KERRGELFYWMH
QQMIARYDAERLSNDMAPTSVFENFDEPIEEGYASDLTVEHTGYRYMFRPEGLTLHDLP-
-ELTKNQLRLWRIRILNAINKGYAY--DKEG---HKVPLNNK----------DGINIISN
FVESSVDSINRPFYGNIHNYAHVIASRIADPDGKYGEDNGVMYDVSTSARDPLFYSWHKF
INSLFIEHKDMLPAYTHDDLE--FKDVVVDTISVKGKEE---------------------
------------------------------TIPNILKTFYRYRTIPLPTGFTFNHEK-KV
DVEHRYLDHEEFEYRLDITNNAAADKHAIIRLYLAPLHHNDGTEYTLTEKRTAVIELDTF
LTTLKPGSNHI-THDSTDSAVTYFHE-------------------KDQHHDC----PCGW
PQNLLLPKSNYQGMEWRLFAVVDDADEEHSE-I------------GENC--NC--GSALS
YCGIVSGKYPNSKAMGYPFDRHIDAH--NVEDW--V--TPNIQHVDITIKFAGK------
---------------HA-----------
>HauHcB1
M-RS-------LIVFCGLLAVAFA-------------------TEN-C-GE-KTDVEAKQ
AKVKEVIMHINRPHVHAGG-----SCE------------PP-PTKLSA--ESLKEIGHLD

```
TNSVFSLFDERNWPEAKEALTLMMKA--ATFEDFITVAKDLSHHMNDDMFLFVFNVAIVH
RDDTSGVRPEFHFKTFPDKYIRHDQIVEMRHAV-------ECGK---DNIKVLANP--HV
FNDTDDAHKLLYWLEDLGLNAHHYHWHTVHPAIWTE-EL-----GRG-KERRGELFYWMH
QQMIARYDAERLSNDMAPTSVFENFDEPIEEGYASDLTVEHTGYRYMFRPEGLTLHDLP-
-ELTKNQLRLWRIRILNAINKGYAY--DKEG---HKVPLNNK----------DGINIISN
FVESSVDSINRPFYGNIHNYAHVIASRIADPDGKYGEDNGVMYDVSTSARDPLFYSWHKF
INSLFIEHKDMLPEYTHDDLE--FKDVVVNTVAVKGKEE--------------------
-----------------------------TQENILTTFYKYRSFALPTGFTFNHEK-HV
EIEHRHLDHEEFEYKLDITNNAATDKHAIIRLYLAPLHHNDGSELTLTEKRTAVIELDTF
LTTLKPGHNQV-THDSTDSAVTYFHE------------------KDQHHDC----PCGW
PQNLLLPKSNYHGMEWRLFAVVDDADEEHSE-I------------GENC--NC--GSALS
YCGIVSGKYPNSKAMGYPFDRHISAH--SVEDW--V--TPNINYADITIKFTGK------
---------------HA-----------
>HanHcB
M-RS-------LIVFCGLLALAVA-------------------SDN-C-GE-KADVEAKQ
AKVKEVIAHINRPLVHAGG-----SCE------------AHPTSHIDS--KTYKEIGRLD
GNSVFSLFDERNWPEAKQAVTLMMKA--ETFDDFITVSNDLSHHMNEDMFLFCFNVAIVH
RDDTSGVRPEFHFNVYPDKYIRHHQIVQIRHEV-------ECGK---DNIKVLANP--HV
FNDTDDAHKLLYWLEDLGLNAHHYHWHTVHPAIWTE-EL-----GRG-KERRGELFYWMH
QQMIARYDAERLSNDMTPNPVFENFDEPIEEGYASDLTVEHTGYRYMFRPEGMTLQDLP-
-ELTKNELRLWRYRILNAINRGFAF--DDEG---NKIELNNN----------DGINIISN
FVESSFDSINRKYYGNIHNYAHVIASRIADPDGTYGEDNGVMYDVATSARDPLFYSWHKF
INSLFIEHKDMLPSYTHDDLE--FKDVVVNTVAVKGKEE--------------------
-----------------------------TVENILTTFYKYRTVPLPTGFTFNKDK-HV
DVEYRHLDHEEFEYKLDITNNAAEDKKAIIRLYLAPLHHNDGSLLTLAEKRTAVIELDTF
ITTLKPGHNQV-THDSTDSAVTYYHE------------------KDQHHDC----PCGW
PQNLLLPKSNYHGMEWRLFAVVDDAEEEHSE-I------------GENC--NC--GSALS
YCGIVSGKYPNSKPMGYPFDRYIDAH--SVEDW--V--TPNINYVDITIKFTGP------
---------------HA-----------
>ScuHcB
M-RT-------LFVLCALVAIAV--------------------AEK-C-SAPSGDVAAMQ
AKIDEVVAPINRPFGHPGG-----DCHH--------------VAPLDA--EKMKGIGLMS
HHEVFSLFDERQWPEAVRVLKLLLEA--ESFDDFIATANSLSHNINEDMFFYCFTVAVNH
RKDCGGLRAPHIYKVYPDKFIRSTYINEIKQQI-------DCGK---DNIKVLVNP--HF
FNNTDSAHQLLYFLEDGGLNTHHFHWHAIHPAIWED-SL-----GRH-KDRRGELFYWMH
QQMIARYDAERLSNGLPPAPTFENFDEPIEEGFAPDLTIDHTGYRYMFRPEHMVLRDLP-
-ELTKNQLRLWRVRVLNAIHAGHAH--HKNG---EEVQLLNE----------NGIDTLAK
FVESSAESINRPYYGNIHCYAHVIASRIADPDGKFGEDNGAMYDVTTSARDPLFYSWHKF
INSLFVEHKDMLPVYSKTELQ--WNDIVVKKVHVKGRET--------------------
-----------------------------GVEDELHTYWKTITAPIQKGFSFTRDK-NV
EIDVRYLDHEEFDYTIDLTNNAAAPKKAVVRIFMAPEHHLDGSMFTPNEMRLNIIQLDAF
TTELTPGANTI-VWKSEMSGVSYHHV------------------QDKNDEC----ICGW
PHNLLLPRGNHEGLDFKLFVVVS---EGSIE-E------------EDTC--HC--KSALS
YCGAVYGKHPSNKALGYPFDRRIDDH--TIEEF--V--TSNMGMTDITIKYTGE------
---------------KH-----------
>SmuHcA
M-RC--------FVLVLLIAATF--------------------ASQ-C-PEPPSDAAAKQ
QQIYQIVEYINKARTPEPI----------------------VEGVDE--SELKYLGHLP
KNEIFSLFDERNWPEAKAAVKYLLEP--KTFDDFIKAAKILITRINDEIFLYALSVAILH
RPDTRGVQVPRIQDVFPDKLIPKFVLQQIREAA-------IRGD---QNPIVYLN---VS
SNNLNVENRLGYFTNDLGMNSHHYHYHVVHPATGVP-IQ-----TVI-KDRHGSRFAHMH
SQIVRRYESERLSNSLPLTESFVNWDEPIQTGFSSHLSIFMKGYNYNFRPEKVRLSNLP-
-ELTKNMMRLWKSRLLYGIHSKSLT--SANN---KNVSLERG----------DGEDLLGN
AIESSLLSINRVFYGNLHNYAHVIAGRASDPDGRYGAHNGVMHDVATSARDPLFYRWHKF
IESIFQQYREYLPPYSHYKLT--YPAVVVEKVEVVPDKR--------------------
```

-----------------------------PDIVNVVRTGYTTNKLKLNPGFDFTRQS-TA
TVNVQHLNHEPFHYKFRIHNKAQQVQNAAVRIFLAPKYDEQGHRLKVNDQRQLMIELDKF
VVHLQPGVNQF-ERSSSESSVTMPTKYFFGDIQD-----------IARNHSC----SCGW
PEYLLLPRGTYEGLKFQLFVAVTNWDEDKVKDK-----------VEPEC--QC--SESLS
YCGTVSGKYPDSRPFGYPFERPIVES--NWEDF--V--TQNIHISDVNIRFVGNA-----

>SdeHcA
M-RC--------FVLVLLIAATF--------------------ASQ-C-PKPPSDAEAKQ
QQIFQIVEYINKAQTPEPV-----------------------VEGVEE--KELKYLGHLP
KNEIFSLFDERNWPEAKAAVKYLLEP--KTFDDFIKAAKILITRINDEIFLYALSVAILH
RPDTRGVQIPRIQDVFPDKFIPNFLLQQIREEA-------IRGD---QNPIVNLN---IS
SNYLNVENSLNYFTNDLGMNSHHYHYHVVHPATGVP-IQ-----TVT-RDRHGSRFAHMH
SQLVRRYESERLSSGLPLTESFVNWDEPIQTGFSSHLTIFKKSYYYNFRPEGITLSDLP-
-ELTKNKMLLWKHRILLGIHSQFLI--SANN---KNVSLERE----------DGEDLLGN
AVESSLLSINRQFYGNLHNYAHVIAGRASDPDGRYGAHNGVMHDVATSARDPLFYRWHKF
IESLFQQLREHLKPYSHYELT--YPAVVVEKVEVVPDKH---------------------
-----------------------------PETVNKVRTGYTTNKLKLNPGFDFTRQS-TA
TVNVQHLDHEPFHYKFRIYNNAQQVQNAAVRIFLAPKYDEQGHRLKVNDQRQLMIELDKF
VVHLQPGVNQF-LRSSSESSVTMPTKYFFGDVQD-----------IARNHSC----NCGW
PEYLLLPRGSYEGLDFQLFVAVTNWDEDKVKDK-----------VEPEC--QC--SESLS
YCGTISGKYPDRRPFGYPFERPIVES--NWEDF--V--TQNIHISDVNIRFVGNVEEEKQ
IE------------KHH-----------
>NadHcA
M-KL-------LILVLALLGSTLAI----------------------C-PDAPGNVKSQQ
EQLQKIVDGVNR-YQPGGQ-----SG----------------PANIDL--QKYYALGHLD
QDDIFSLFDERNAEEVRQSIQYLIEP--SSFDGFIQAATQLSHIVNDELFLYALSVAVLR
RPDTAGVHVPRIHDVFPDKFIPRDILQNIRESA-------ITKS---EQAEYYYNP--VT
RNYLNVEDRLNYFTDDLGLNSHHHHYHVLHPPTGFPGQS-----HSD-SDRHGEFFYWMH
HQFVARYEAERLSNGLPLTEPFENWDEEIHTGYAPHLTVFKKGYNYNFRPEGLVLSDLP-
-ELTKNKMRLWKSSLLYAIQVNQMI--AANG---SSVDIHNS----------HGIDVVTN
AVVSTNHTINRPLYGNLHNYAHVIASRVSDHDGRYGVEGGAIEDVVTSARDPLFYQWHKF
IDEILQEHKDSLHPYSQYQLT--WSDIELKDASVVPAAR---------------------
-----------------------------PDEPNVIHTYYSSSRYQIRQGFDFTRDR-AA
TINVNHLNHEPFKYRLRIRNNAQEPRTAAVRIFLAPKYDEQGHRFEVNEQRKLMIELDKY
VLTVQPGEQNY-LRSSAVSSVTVPRSYIYDDVQK-----------VPHTHRC----ACGW
PEYLLLPRGSYEGMEFELFVAVTDWNEDQARGN-----------VQPEC--QC--SQAWS
YCGSLQGTYPDSRPLGYPFDRHVEES--SWEDY--L--PHNIQHRDVVIQFVGYQNPHQE
--------------NNQ-----------
>SmuHcB
M-AA----LQWFLLGLVALSLVA--------------------GEQ-C-PK-TNDIQEKQ
RRLVEALSFVNRPFNPKSK-----DHDL--------------TSAEKQ--FLDSHVGYLP
RREVYSVFDARYLPEANASLYFLLDA--PNFDGFIRAVDILSHRINEDMLYYVVSVAATH
RDDTRGVILPRIHDLYPDKVLTHDIIVQIKQKA-------LRND---KDIVVDGTK--QH
VDYRNPYSRLHYFLNDIAMNSHHYHWHVQNSLIWNN-RTYPSVANLL-KERVGAAFAYMH
HEMVNRFDAELLSNYLPRVTPFENWNDPIPEGYASHLTVDRYQYSYMFRPSNLPLEDLS-
-DITRNEMRQWRDLILNAIHKGYAI--TKDG---RNLTLSEE----------DGIDIIGN
MVESTVNSINLPLYGNLHCYAHTIAARVADPDNTYGEDNGAMYDVATSARDPLFYRWHKF
IDNLIQEYRNSLRSYTNEQLK--WSVVVVEGLTVEG------------------------
------------------------------AKVNKIKTFWEDDVLTVGTGFSFTGPSATA
KVNVRHLQHEEFSYNIQVVNNAGENKKAVFRIFMAPKYDEKGYEYDFNEQRQSMFEMDKF
VKELTPGKNVV-VRKSSESSVTQKHEKIYSNPKE-----------RQQNDHC----SCGW
PDNLLVPRGSYEGTEFQVFVVVTNYEEDYVP-S------------DESC--HC--GDGRS
YCGILFGNHPDRRTYGYPFDRKTNAH--TFPDF--K--TPNMNAVDVSIQFTGVIK----
--------------KSS-----------
>SdeHcB

M-AA----LQWFLLGLVALSLVA--------------------GEK-C-PK-TNDIQEKQ
RRLVEALSYVNRPYNPKPK-----DHEL--------------TPAEKK--FLESHVGYLP
RREVYSVFDARYLPEANASLYFLLDP--PNFDGFIRAIDILSKHINEDMLYYVVSVAATH
RDDTRGVILPRIHDIYPDKFLRSEIIGQIKQKA-------LGNE---KDIVVDDTK--QH
VDYRDPYSRLGYFLNDIAMNSHHYHWHVQNSLIWKN-RTYPSVANLL-KERIGAAFAYMH
HEMVNRFDAELLSNKLPRVTPFENWNDPILEGYAAHLIVDRYQYNYMYRPPNLKLKDLP-
-ETTRNQMRQWRDRILDSIHKGYAI--SKDG---RNVTLSEE----------DGIDIIGN
MVESTVNSINLPLYGNLHCYAHTIAARVADPDNTYGEDNGAMYDVATSARDPLFYRWHKY
IDKIIQAYRNSLRSYTVEQLT--WPVVVVEGLTVEG------------------------
------------------------------AKVNKIKTFWEDDVLTVGTGFTFTGPSATA
KVNVRHLEHEEFSYNIQVVNNAGENKKAVFRIFLAPKYDEKGHEFDFNEQRQSMIELDKF
VTELTPGKNVV-IRKSSESSVTQKHEKIYANPKE-----------RQQNDHC----NCGW
PDNLLVPRGSYEGTEFQVFVVVTNYEEDYVP-S------------DESC--HC--GDGRS
YCGILFGNHPDRRPYGYPFEKRTDAH--TFQDF--K--TPNMYAVDVSIQFTGVIK----
--------------KSS-----------
>SbaHcB
M-AA---SIAWFLLGLAAVSVIS--------------------AEK-C-PK-TSDIEAKQ
QRLVQVLSYVNRPYNPLPK-----KHAL--------------TPEEEN--LVRNHLGVLG
RREVFSVFDERYWPEANAYLQYLLAV--ESFDDFVQAVEVLGHSINEDMLYYVVSVAAVH
RDDTRGVVLPRIHDVYPDKFLPTEVIVQIRERV-------IKNE---KDIVVNATE--QH
YDYRDPYSRLRYFLEDVGMNSHHYHWHVQNSLLWKN-QKYPSAANTL-KDVSGAAFVHMH
QEMVNRFDAELLSVGMPRVTPYENWDEPIPEGFSSHLTVDRYQYRYMFRPANLILSDLP-
-EMTRNQMRQWRDRVLQAIHKGYVI--TSYG---RNVSIHGE----------NGLEILGN
IVESTVNSVNRPLYGNLHSYAHVIAARVADPDQRYGEDYGAMYDVTTSARDPLFYRWHKY
MNNLFREHSNSLQPYSIQQLI--WSDVTVEGVTVEG------------------------
------------------------------SKVNKIKTFWEDDVLTVGSGFSFTGQS-KA
TVKVRHLEHEEFTYNIQVTNGAESSKKAVIRIYLAPKYDEQGHEFDFEEQRQSVIELDKF
VYELTPGKNTV-SRKSSESSVVQKHERVYANPEQ-----------RQQNDHC----SCGW
PDNLLVPRGSYEGTVFQVYVVVTSHEEDYVQ-S------------DESC--HC--GEGRS
YCGILLGANPDRRSFGYPFDRRTHAH--TFHEF--K--TQNMNVADISIQFTGTIK----
--------------KSS-----------
>AgrHcB
M-VG----------LAVLIGVVA--------------------GEK-C-PK-TSDIQTKQ
KQLQEILSYVNRPYVTLSKQ----QHSL--------------TPEEEV--YLRNHVGHLA
KREVYSVFDERYMEEANASLHYLLEP--QSYEGFVRAAEILRHHINEDMFYYVFLVAATH
RDDTRGVVLPRIHDVYPDKFLWTDVIVQIKQKA-------IRNE---QNIVVDATE--QP
YDYRDPYSRVRYFLEDLALNSHHFHWHVQNSLLWKN-EPHSSAAGTP-KDRSGENFYWMH
HQMVNRLDAELLSSSMPSVTPYENWDDPIEEGYASHLTVDRNQYRYMFRPANLVLKDLP-
-EMTRNQMHQWTDRILLAIHKGYVI--TRDG---KNVTLRGD----------NGIDILAH
IVESSVNSVNRLLYGSVHTYAHVIAARIADPDQKYGEDYGAMYDVSTSARDPFFHRWHKK
VDNVLQQYKSSLRAYQPQQLT--WNDVVVEGVTVEG------------------------
------------------------------SKVNKIKTYWEDEVVSVGSGFSFTGQS-TA
RVNVRHLQHEDFIYNFQITNNAGSNKKAVFRVFLAPKYDETGHEMDFNEQRRSVIELDKF
VHELTPGKNAV-TRKSSDSSVVQRKGQVFVTPEQ-----------RQQSDQC----SCGW
PAHLLVPKGSCEGTVFQVYVVVTNYDEDYVE-S------------DQSC--HC--GEGRS
YCGVLLGENPDRRPLGYPFDRRTDAR--TFEEF--K--TQNMNIADVSIQFVGTVK----
--------------KSG-----------
>TspHcB
M-AI---QPVWVFAAFAILSFVA--------------------AEK-C-PQ-TSDIQTKQ
QKLVEILSYVNRPYVKWNRE----KQPL--------------NPQDEE--ILHGKVGLLN
QREVYSVFDERYWGEANASLYYLLRP--QSFNDFVKAAEVLRQYVNEDLFHYVFLVATTH
REDTRGVVLPRVHDSYPDKFFKTDVITQIKAAA-------LLGR---KDIVVDDTE--ED
YDYRNPYSLLSYFLEDLGMNSHHYHWHVQNSLLWSN-RLQRGGAQTLQKDRYGEAFYWMH
HQMVNRFDAELLSNRLPRVTPYENWDDPIEEGYASHLTVDRHQYRYMYRPPGLVLRDLP-
-EMTRNQMRQWRDRVLVAIHKGYVT--TRDG---RNVTLNDP----------EGIDILSH

```
LLESTVESINRPLYGNVHSYAHVIAARVADPDQRYGEDYGAMYDVTTSARDPLFYRWHRY
VDNILQSYKNTLPHYTENDLL--WNDVKVESVTVEG------------------------
------------------------------SKINKIKTYWEDETVTIGKGYSYTGDS-SA
SVKIRHLQHEEFNYNIQINNNAGSNRRAVIRVYLAPKYNEKGHELSFEEQRRSVIELDRF
SQELVPGKNTV-TRKSVESSVTQSNEQIFADPQR-----------RQQQDHC----SCGW
PNYLLVPRGSYEGTEFQVYVVVSNYDEDHVE-S------------DQSC--YC--GQGYS
YCGVLYGKYPDRRPFGYPFDRQSDAR--SFDEF--K--TQNMVATDVSIQFTGNIK----
--------------KSS-----------
>NadHcB
F-YSP--TISWIFLAIAIFTLAS--------------------AEQ-C-PR-TKDIDAKQ
GQLLDIIKYVNRPYHQLPQR----DQNL--------------SPDEEK--KYRSHWGILE
QREVYSVFDERYIEEANASLHFLLEP--DSYDQFIRRAEILRHYINEDMFLYVLNVAVTH
RDDTRGVVLPRIHDVYPDKFLTTDVIVQIKEAV-------LRGE---KNIVVDDTA--HD
YDYRNPYSRLNYFLNDLALNSHHYHWHVQNSLLLGN-RVIPSGPDTS-KDRTGEAFYWMH
HQMVNRFDAELLSNHLPRTTPFENWDDPIEEGYAPHLTVDRYQYRYMFRPANLVLRDLP-
-EMTRNQMRQWRDRLLNAIHRGYVI--SSSG---ANVTLRDE----------NGIDILSH
LVEPNVDSVNPNLYGGLHSYAHVIAARVADADHKYGEDYGAMYDVVTSARDPLFYRWHKY
VDDVFQEYKDSLHPYTEEERN--WNDVKVEEVTVEG------------------------
------------------------------SKVNKIKTHLEEDIVHVGSGFSYSGDS-SA
KIKVRHLAHEDFKYNIQVTNNAGSAKRAIIRIYLAPKYDEQGEQIEFNEQCRTVIELDKF
AAELAPGKNTI-TRQSSESSVIQDHKNVYADPSS-----------RQDDDSC----HCGW
PDYLLIPRGSSEGTEFQLYVVVSDYQKDGID-V------------DQSC--HC--GDGHS
YCSVLYGQNPDRRPHGYPFDRHTDAR--TFEQF--R--TPNMYQTDISIRYDNTVVK---
--------------GSS-----------
>ChoHcB
M-GH----LTWLLVCALAIGLAS--------------------SEK-C-TS-TGDIRAKQ
QKLTEIIQYINRPYNIQTP-----KPQP--------------TPEEEE--YFRQNVGYLD
EHEVFSVFDERYWKEANASLHYLLSA--NTYQDFVRAAEILRYYVNDDLFHYVFLVAVTH
RDDTRGVNLPRVHEVWPDKFWHRDVISELKLAA-------IHSQ---KDIYVNDTE--IR
HDYRDPYSAVSYYLEDLGLNSHHYHWHVQNSLLWDN-EDWPSVSKVH-KDRLGEAFYYMH
NQMVNRFDAELLSNHLPRVSPFENWDEPIEEGYASQLSIDSIQYHYTYRPPRIVLQDLP-
-EMTRNQLSQWRSQILQAIHHGKVV--RDDG---ETIYLTRN-----QPGTDQGIDVLGH
LVESTVQSANRPLYGNIHSYAHVIAARVADPDSRYEAANGVMYDVTTSARDPLFYRWHKY
VDDILQEYKNSLEPYSHEDLS--WNDVAVEQISVEG------------------------
------------------------------SRVNKIKTYWEDESVQLQNGHTFTINT-TP
KIRTRHLAHEDFSYNLQVTNNAGGETKAIIRIYLSPKYDEQGEPLDFNDQRQSVIQLDKW
VQTLTPGKNII-TRKSVESSVTQKHSDIYSHYTS-----------QENDDHC----NCGW
PSNLLIPRGSWEGTQFTVFAVVSSWEEDHVQ--------------GEGC--QC--GDGTS
YCNVLLGKYWDRRPFGYPFDRHSDAR--TYEDF--R--TPNQNSVDISIQFVGNVK----
--------------KSS-----------
>ScoHcA
M-WS-----LALATLFVLGTVIR--------------------ADQ-C-PPVPADTKDKL
EKILELIGHVNRPLTPETP-----EP-----------------AGYDE--TKLKGLGILP
QHEIFSLFDERTWPEATKAAEFLMEA--TDFEHFIQRADVLRHRINEDMFMYALNVAVLH
RKDTRGVQVPRIHKIYPDKFLKQDILVEVREKV-------NHGE---EKPVVDATE--LH
QNQLDPNYRLSYFLEDIGMNSHHYHWHVVHPAVWLP-KH-----GPR-KDRKGELFYYMH
HQMVARYDSERLSNNLPRTEPFENWDDPLEEGYAPHLTIHKTGYNYMFRPEGLIVRDLP-
-ELNKNKMRQWKSRILHGIHLNVLY--AENG---TKISLDNE----------HGIDLLGD
AIESSLLSVNRAFYGNIHCYAHVMAARIADPDGRYGEDNGVMHDVATSARDPLFYRWHKF
IDNIFLEYKDNLDPYTQYELT--WPDVVLNDVTVKPHKG---------------------
------------------------------DYDDEVHTYWEVDNYELGKGFDYTRKT-TA
TVKVRHLQHEDYHYEIDIDNNAGKAKKAVFRIFLAPKYNEKGELFPVNEQRQLLVELDKF
VATLEPGHNVI-ERQSKESSVTMSKDHVFGEIRN-----------LADDHQC----SCGW
PDYLLLPKGKYEGMTYQLFVVATDYEEDHVEDA------------GEEC--QC--RDSMS
YCGSVEHKLPDNKPLGYPFDRRIDGT--GFEEF--K--TQNMYYGDVVIQFTGETV----
```

---------------TH-----------
>SweHcA
M-WT-----LAIATLLIVGTAVS--------------------ADK-C-PPVPADTKAKL
EKILELVGHVNRPLTPETP-----EP-----------------AGYDE--AKLKGLGILS
NHEIFSLFDERLWPEAMRATEFLMEA--TDFDHFIQRADVLRHRINEDMFMYALNVAVLH
RKDTFGVQVPRIHKIYPDKFLKQDILVEVRERV-------NHGE---EKPVVDATE--LH
QNQLDPNYRLSYFLEDVGMNSHHYHWHVVHPAVWLP-KH-----GPR-KDRKGELFYYMH
HQMVARYDAERLSNNLPRTEPFENWDDPLEEGYAPHLTIHKTGYNYMFRPEGLIVHDLP-
-ELNKNKMRQWKSRILHGVHLNKLY--AENG---TEIDLDSE----------HGIDILGD
AIESSLLSVNRPFYGNIHCYAHVMAARIADPDGRYGEDNGVMHDVATSARDPLFYRWHKF
IDNIFQEYKDNLAPYTQYELT--WPDVVLHRVTVKPHKG--------------------
------------------------------DYDNEVHTFWEVDKFDLLKGFDYTRQT-TA
TVKVRHLQHEDFHYEIDIDNNSGKSKKAIFRVFLAPKYNEKGELFPVNEQRQLLIELDQF
VATLEPGNNVI-EHQAKESSVTMSKDHVFGDIRT-----------LSDDHQC----SCGW
PEYLLLPKGKYEGMTYQLFVVATDYEEDHVKDA------------EEEC--LC--RDSMS
YCGSVESKMPDSKPLGYPFDRRIDGT--GFEEF--K--TQNMYYGDVVIQFTGETV----
---------------DH-----------
>SguHcA
M-WS-----LAIATLLVLGTVAR--------------------ADE-C-PPVAGDAKDKL
EKILDLLGHVNKPLTPETP-----EP-----------------AGYDA--AKLKGLGILP
NHEIFSLFDERTWPEAMRAVEFLMEA--EDFNEFMQRADVLRHHINEDMFVYALNVAILH
RKDTRGVQLPRIHKIYPDKFLKHDILVEIREKV-------NHGE---EKPIVDATE--LH
QNQLDPNYRLSYFLEDIGMNSHHYHWHVVHPAVWLP-KH-----GPP-KDRKGELFYYMH
HQMVARYDAERLSNNLPRTEPFENWDDPLEEGYAPHLTIHKTGYQYMFRPEGLIVRDLP-
-ELNKNKMRQWKSRILHGIHLNSLY--AENG---TKIILDSD----------HGIELLGD
AIESSLLSVNRAFYGNVHCYAHVMAARIADPDGRYGEDNGVMHDVATSARDPLFYRWHKF
IDNIFQEYKDNLRPYTQYELT--WPDVVLNRVTVKPHKS--------------------
------------------------------DYDNEVHTFWQVNNYELGKGFDYTRQT-TA
TVKVRHLQHEDFHYEIDIDNNSGKNKKAVFRVFVAPKYNEKGELYPVNEQTKLFIELDKF
VADLQPGNNFV-KRESKESSVTMSSDHIFGDIRG-----------LAHDHQC----SCGW
PDYLLLPKGKYEGMTYQLFVVATDYEEDHVQDA------------GEEC--QC--RDSMS
YCGSVEHKLPDNKPLGYPFDRRIEGT--GFEEF--K--TQNMYYNDVIIQFTGETV----
----------------V-----------
>ScoHcB
M-VA-----KWCVLAMCLLVAVG--------------------ADK-C-PR-ATDQEAKQ
KRMLEVLQHVNKPYVAETK-----DPK---------------IPAGSE--DVFKHLGILE
KHEVFSLFDERQWDEATTAAVYMLTA--PSFDEFIDRAEIVRHRINEDMFYYAFSVAAVH
RDDTRGINLPRIHEIYPDKFLKHKVIVEVKNSI-------NSGQ---EDPLIDATH--EF
TDLRDPNSKLHYFLEDVGLNSHHYHWHVIHPAVWQE-SL-EELTHQH-KDRKGELFYFMH
HQMVNRYDAERLSNGLPRSTTFENWNDPIETGYAPHLTIDRTGYRYQFRPDNLVVRDLP-
-ELTKNHMRQWRDRILYAVHRGEAL--AANG---SSVSLRDE----------RGIDVLGN
MVESSLQSINRPFYGNVHCYAHVIAARIADPDGKYGEDNGAMYDVATSARDPLFYQWHKF
IDNLFHEYKDALKAYSSEDLT--YNDITIEEVNVQGEGG--------------------
------------------------------SPANTVTTFLENSIVHLDEGFSFTARG-HA
RVKVQHLQHEGFNYQIKV-NNAGGEHKVVFRVFLAPKYDEEHHEFDFNEQRGMAIELDKF
VATVPAGSSTV-EQHSSKSSVTQSNDNFYGSSAT-----------RSSENHC----SCGW
PDYLLIPKGNHQGVQFNVYVIATSYDEDHVE-S------------DESC--HC--GDSLS
YCGALYDKYPDRRPMGYPFDRHADAQ--TFDEF--K--TKNMNSVTVTIKHTGEV-----
--------------KDA-----------
>SweHcB
M-AT-----MWCVLAMCLLVAVG--------------------AEK-C-PR-ADDQEAKF
KRMAEVLHHVNRPYVLETK-----DPH---------------IPAGSE--DVFKHLGVLD
KHEIFSLFDERQWDEATSAAVYMLTA--PNFDEFIDRAEIIRHRINEDMFYYAFSVAAVH
RDDTRGISIPRIHDVYPDKFLKHKVIVEVKNKI-------NGGE---ADPIVDATH--DF
TDLRDPNSKLHYFLEDIGLNSHHYHWHVIHPAVWQE-SL-EELTHQH-KDRKGELFYFMH

```
HQMVNRYDAERLSNGLPRACTFENWNDPIEEGYAPHLTIDRTGYRYQFRPDHLIVRDLP-
-ELTKNHMGQWRDRILYSVHRGSAL--AANG---SRVSLRDD----------RGIDILGN
MIESSLQSINRPFYGNVHCYAHVIAARIADPDGKYGEDNGAMYDVATSARDPLFYSWHKF
IDNLFHEYKDALKAYSHDDLE--YKDVTVSDVSVHIEGS---------------------
------------------------------NVENEIKTYWENNVLHLADGYSFTARG-QA
RVKVQHLQHEDFDYFIKL-NNAGAEQEVVFRLFLSPKYDEENHEFDFNEKRKMAIELDKF
TYLVSAGESTV-VRHSVDSSVTQRIDNFYGDVAS-----------RKTDNHC----SCGW
PDYLLVPKGNYEGMKFDLFVVATSAAEDVVE-S------------DKSC--HC--ADSLS
YCGTLYDNYPDKRPMGYPFDRHSDAH--TFEEF--K--THNMNSATVTIKFTGEV-----
--------------KDA-----------
>SguHcB
M-AA-----MWYFVAISLLVAVG--------------------AEK-C-PK-ATDPEPKL
KKMLDVLHYVNRPYVVESK-----DPS---------------VPAGSA--AFFKHFGILD
KREVFSLFDERQWDEATSAAVYMLTA--PSFDEFIDRAELVRHRINEEMFYYAFSVAAVH
RDDSRGISLPRIHEVFPDKYLKYSTIVDVKNKI-------NSGQ---TDPLVDATH--QL
YDLRDPNSKLHYFLEDIGLNSHHYHWHVIHPAVWEE-SL-EKLTGQH-KDRKGELFYFMH
HQMVNRYDAERLSNGLPRAVTFENWNDPIAEGYAPHLTIDRTGYRYQFRPDNLIVKDLP-
-ELTKNHMRQWRDRILYAVHRGEAL--AANG---SHVSLREE----------RSIDILGN
MIESSLQSINRPFYGNVHCYAHVIAARIADPDGKYGEDNGVMYDVATSARDPLFYQWHKF
IDNLFHEYKDALKPYTKEDLSKGVENISVDEVTVVGDGG---------------------
------------------------------SPANTVTTFLQNSIMHLAEGYSFTARG-TA
RVKVQHLQHEGFKFQIKV-NNGGGERKLIFRVYMSPKYDEEHHPFSFNDQRHMAIELDKF
VATVASGDTTV-ERHSSQSSVTQSIENFFGDPNT-----------RKTDNHC----SCGW
PDYLLIPKGNYEGVQFNVYVMATSYDEDHVE-S------------DESC--HC--GDSLS
YCGALYDNYPDKRPMGYPFDRHADVH--TFDEF--K--SKNMNLGVVTIKYTGEV-----
--------------KDV-----------
>PanHcBI
M-KL-------ILCLTLFIGVAL--------------------ADRNC-PA-PTNVEAKQ
AMVDNLLTFINKPDNPPAP-----------------------VPNVDP--SKLKGVGYLP
KREVFSMFDEREWPEAIEVLKRFLTP--PTFEEFIKVAETLYPRMNEDLFFYCFSVAIVN
RPDAQGVRVPRVQDVYPDKFFEHYLLVSIKDKI-------MTGH---KNPVVNDTH--DF
HNHYDPYRRVDYFTEDMGMNSHHYHWHVTHPLFLPD-VI----EGVH-KDRIGELFYWMH
RQMVARFDSELLSNHLPRVSAFEHWDEPIHTGYAPHLTIHRTGYRYLYRPDGLILQDLP-
-ELTKSQMQQWKLRILKAIKRSNYV--ATNG---SYVALDKV----------NGIDTLAH
LIISTTGSVNRRYYGNLHSYAHTIAGKIADASGKYAEDAGVMIDVTTSARDPIFYQWHKY
IDGLFQTYQKTLKPYNKYELS--FPGVSIEDVHLETESD-RKSEHHDDHHDD--------
--------------KHDDKHNESHDDHHDDHHEDVIHTYWTLNDFKLTKGFDYTLDS-EA
IIHMKHIDHEDFTYVFDVVNHDQKEKHAAIRVFLRPTYDEAGHEFTPEQFWPLIIELDKF
TYTLKPGHNII-RHNVNESSVTMPREQIYDSVKQW---------STKEHDHC----HCGW
PEYMLFPKGNYEGMAFKLFVMVTDWDQDKIS-D------------DTSC--HC--KNSLC
YCGTIFQEYPDKRPMGFPFDRPLPKS--GWEAF--R--TPNMFRKDLTVKFTGYSH----
--------------IKH-----------
>PseHcBI
M-KL-------VICLTLLISVAL--------------------AEKHC-PA-PTNVDAKQ
AMVDNLLTFINKPDNPPAP-----------------------VPDVDP--AKLKGIGILP
KREVFSIFDERMWPEAIEVLKRFLTP--ATFEEFIKVAETLYPRINEDLFLYCFSVAIVH
RPDAQGVRVPRVQDVYPDKFFEHYLLVNIKDKI-------MSGK---KNPILNDTH--DF
HNHYDPYRRIDYFTEDMGMNSHHYHWHVLHPMILPN-VI----SGRT-KDRVGELFFWMH
RQMVARFDSELLSNHLPRIDAFENWNEPIHIGYAPHLTVHRTGYRYMYRPDGLVLQDLP-
-ELTKHQMKVWKSRILKAIGRQNYV--AKNG---TDVAIDPV----------HGIDTLAH
LIISTTDSVNKMVYGNLHSYAHTIAGRIADAIGKYDEDSGVMSDVTVAARDPIFYQWHKY
IDVLFQTYQKTLLPYKSHEVG--FPGVTIEDVHIETENDLHKDDHKDDHNND--------
--------------HKDDHDNDSHHEAHHHDHDDVIHTYWSTNDFKLSKGFDFTLDS-EA
IIHMKHIDHEDFTYVMDVVNTDAKEKHAVVRVFLRPKYDEFGNKFTPQEIWPMMIELDKF
TYTLKPGHNII-RHHSNDSSVTMPREQIFESAKHW---------STKEHDHC----HCGW
```

```
PDYMLLPKGNYEGMEYYLFVMVTDWDQDKVS-D------------DTSC--HC--KNSLS
YCGTIFEEYPDKRPMGFPFNRPLPKG--GWEAF--R--TPNMFRKSLTIKFTGYSH----
--------------LKH-----------
>ChuHcBI
M-KV-------LICVALLVAVTL--------------------AEK-C-PV-TIDLQAKQ
DAINRLLKSVNKPFNPKVSV----------------------PPMPDD--VSNIKLGVLP
KREVFSIFDERTWPEGIEVVKRTTNSKRLLYEFNLNKAEILYPRINEDLFFYCLSVAIVH
RLDTQGLKVPRIQDVYPDKFFEHQLLVKIKDEI-------MTGH---KNPELNDTH--DF
HNHYDLYRRIDYFTEDFGMNAHHYHWHVVHPMFKPD-VL----PGGD-KKRSGELFYWMH
RQMVARFDSELMSNNIPRIRSFENWNEPIPIGYAPHLTIHRTGYKYGFRPDNMVLRNLP-
-ELTKYEMKQWETRILRSIHKSKIN--QPLG---NSIGLNDT-------------DTLAH
LIMSSTDSANRQYYGNLHSYAHVIAARITDPRNKFREDGGVMSDAAVSARDPIFYQWHKY
VDSIFQAYQKTLKPYRPSELE--WTDVTVNEVYIDATKE---------------------
------------------------------HPRNEIRTFWGMNDFRLTKGFDYTIDS-EA
VIHLRHLDHEPFTYVFDVNNNRNKEREAVFRVYMVPKYDEHRTKFSINKQRPLMIELDKF
VRKLHPGHNEI-RRHANDSSVTMPRELIFDRMAD----------TTVEHDQC----QCGW
PEYMLVPKGKYEGMTFQLYVTVTNYEEDFIETE------------DTSC--YC--RNSLS
YCGTIYQEYPDRRPMGYPFDRPIKAR--CWEDF--K--TDNMYRTDVTIRFTGYT-----
----------------H-----------
>AmaHcB1
M-RL------VILLVALSAAVCY--------------------AAK-C-PQ-PTNVAQKQ
AQMDKILSYINKPLNPAPP-----------------------VIDVDK--TKLKGIGILP
RHEVFSVFDERMWPEAFRVLEKMLAA--QSYDDFIKVAEALYSRINEDLFLYCLSIAIVH
RPDAQGVHVPRVHDIYPDKFLTNDVIHKIKNKI-------IRGM---KNPIVNDTH--GH
HNHYDLYNRVSYFTEDLGMNSHHYHWHVLHPSIWTS-EI-----G-R-KDRDGELFFWMH
RQMVARFDAELMSNHLPRIRPFENWNEPIEMGYAPHLTVQRTGYTYTFRPEGLTLKDLP-
-KMTKSHLRQWRQRILADMHTGLVR--AENG---TKVPLSEE----------RGMDTLAH
MVVSTLGSINRDYYGNLHNYAHVIAARIADPKGKHKEDYGVMYDATTSARDPLFYSWHKF
VDYIFNEYQRTLEPYSKNDLY--WDDVILSDIHVDCHRD---------------------
------------------------------DDHDEIHTFWRTNTVHLEKGFDFTPKS-EA
TVVVRHLDHEDFTYVFEVDNKAKTEKHAVFRVFLAPELDDCGHQLTVEDQRLLMIELDKF
VATLKPGHNVV-RRNSNESSVTMPTREIFDGVEY-----------LVDEDHC----HCGW
PEYMLIPKGNYQGMRYRLFVMASDWDEDKAT-D------------DNSC--HC--KDATS
YCGSIYDEYPDKRPMGYPFDRQVHYY--NWDQF--K--TQNMRSIPIIVKFTGETLPEE-
--------------KLH-----------
>CleHcBI1
M-RF------LICAVALLAAVCY--------------------AAK-C-PA-PTNLDAKQ
GQIDKVLTYFNKPLNPSPP-----------------------VSDYDK--TKLKGIGVLP
RHDIFSIFDERVWPEATKVLEKLLAA--PTFEDFVKITEALYSRVNEDLLLYVLSVALVH
RPDTQGVHIPRIHDIYPDKFLTNDVVHKLKDKI-------VHGK---KNPTVNDTH--DH
INNFDLFHRVAYFTEDLGMNSHHYHWHVLHPGIWNE-KF-----G-H-KDRNGELFYYMH
RQMVARFDAELMSNHLARIKPFEDWSQPIKIGYAPHLTIQRTGYNYMFRPAGLKMRDLP-
-ELTKHQMRQWKHRITTAIHTGYVL--HENG---TRLSLRDE----------HGIDTLAN
LLVSTVESVNHEFYGNLHSYAHVIAARIADPDGKFKEDSGVMADATTAARDPLFYSWHKF
IDNIFAEYQESMPAYTKYDLR--WQDVIIKDIGIDCHEY---------------------
-----------------------------KNEDNIIHTFWTRNTLKLNKGFDFTRDS-EA
IVNVKHIDHEDFAYVFHVDNNGKTDKHAVFRVFIAPEVDERGHHLEVEEQRRLMIELDKF
VYTLKPGHNEI-RHNSNESSVTMPVREIYGNVEE-----------LHDDDHC----HCGW
PEYMLIPKGTHQGMQFKLFVFASDWDEDKAS-T------------DTSC--HC--KDSLS
YCGSIYEEYPDKRPMGYPFDRHVEVN--DWSHI--R--TQNMAYTDLTIKFTGEDRVYEH
N------------HQH-----------
>CleHcBI2
MMRF------VVCAVVLLAALCS--------------------AAK-C-PV-PTNLKDKQ
SKIDKILTYFNKPFNPSPP-----------------------TSDFDK--TKLKGIGVLQ
HGDIFSTFDEQVWPEAIKVLKLLMDA--PTFDDFIKTTEALYSHVNEDLLLYVLSVALVN
```

```
RPDAQGVHIPRVHDIYPDKFITKDVIHELQKLI-------DQGN---KNPIVNDTY--DD
IENSDLNSRVVYFTEDHGMNSYYYRWHVLYPRIWNE-KF-----G-H-KDRKGEHFYYMN
RQMVARYDAELLSNHKARILPFEDWNQPIKIGYASHLAIERTGYHYASRQDGLKLRDLP-
-ELTKRQMRQWKREITTTINTENVF--YKNG---TELPLGDE----------HGIDTLAN
LVAPTVVSINRQFYGNLLSAAHAITARITDPDGKYQQESSVMADAATAARDPLFYSWHKF
IDNLFAEYQKSLLPYTEKELT--WDDVAISSISIESNEY---------------------
------------------------------REEDIIHTFWTRRALKFDKGFDFTMDS-EA
IVNIKHVDHEDFTYVFYVDNYGNADKNAIFRVFIAPEVDEQGKPLKVKEQRKLMIELDRF
VFTLHQGNNLI-RRNSNESSVTKPVQEIYGNMGE------------LDDDHC----HCGW
PEYMLLPRGTRRGMQFNLFVFASDWDEDKAT-T------------DTSC--HC--KDVLR
YCGSIYEKYPDKRPMGYPFDRRMEVN--DWRDF--N--IPNMAIKAFTIKFTAE------
--------------ERV-----------
>TcoHcB1
M-KL------LIIAVALLVTMAY--------------------ADK-C-VP-SPQTEANQ
ALLDHLLTYINKPANPRPP-----------------------VPLVDP--VLLKGIGILP
KHEVFSIFDERTWKEVVVILKGMMDA--STFEEFIKFSEKVYPRVNEDLFLYCLSVAIVH
RLDAQGVDVPRVHDVYPDKFLTNDIIVKIKNMI-------IHGE---KNPVVNGSR--FF
YNDLDPYHRVAYFTEDLGMNSHHYHWHVVHPSIWNK-EL-----GPP-KDRSGELFYWMH
RQMVARFDSELLSTHLPRIHPFESWHDPITIGYAPHLTIQRTGYNYMNRPEGLTLQNLP-
-DLTIHEMNLWHSRILEAIHRHQLH--TKNG---TKIPLDEK----------HGIDLLGH
LVVSTLGSINRKYYGNLHSYAHLIAGRIADVKYKHMEDNGAMYDAATSARDPLFYSWHKF
IDNIFTEYQETLRPYNKYELT--WPEVIIDDFYVEVNST---------------------
------------------------------KTHNLIHTYYTSSTLKLDKGFDYTRDS-EA
KVVIKHMDHEDFSYHIDVDNNAKAPKKAVIRIFLAPKYDEHGHPLTINEQRVLMIELDKF
IETLNPGHNVI-HRSSINSSVTINSKQIFGDRKI-----------YKDNDHC----HCGW
PDYLLVPRGDYDGVHYELFVMASEYEKEKVSDK------------PTSC--RC--KDSLS
YCGTIYEEYPSNKPFGYPFDRVIRVR--GWEEF--K--TQNMFDTDVTIQFTPTF-----
----------------Y-----------
>SpiHcB1
M-KL------FILAVVLLATTAI--------------------AEK-C-PK-PVNVEAKQ
KMVDTLISYINKPANPVPP-----------------------KPPIDP--KKLIGIGILP
KDQVFSLFDERTWDEVSEVLKRFMAA--PTFEDFIKVAETLYPHVNEDLFLFGLSVAIVH
RPDANGVHVPRVHEVYPDKFLTNDVLINIRKQI-------IKGH---KNPVVKDTH--YY
HNDFDPFRRVDYFTEDLGMNSHHYHWHVLHPSIWTQ-DI-----G-E-KSKLGELFYWMH
RQMVARFDSELLSVHLPRISSLDDWNKKVKIGYAPHLTIQRTGYTYMNRPENLEIEDLP-
-ELTKGELMQWKNRIMEAIARHNITLKGGPG---KKLRTTPE----------NGIDIFGH
LIAATKNSTNRRYYGNLHSYAHVIAARIADSDGEHMEDNGAMYDVATSARDPLFYSWHKF
IDKLFTEYQMTLTPYTPYQLT--WPDVVVDGIHIENLKT---------------------
------------------------------HEENMIHTYYTSSTLRLSKGFDYTKDS-EA
KVIVEHTDHDDFVYVIDIDNNARVEKTAVLRIFLAPKYDERGHPLTLKEQRVMMIELDKF
KATLKPGHNVV-RRCSNESSVTMPVDHIYGDITR-----------TVDEDHC----HCGW
PEYLLVPQGDYDGMHYELFVMATNYDEDKAQDT------------DLSC--KC---HAHS
YCGNILGEYLDKRPLGYPFDRKIKAT--GWAEF--K--TQNMCDADIVIKFSGKN-----
--------------KDH-----------
>AgiHcB1
M-KL------FVLAVVLLATTAI--------------------AEK-C-PK-PVNVEAKQ
KMVDTLISYINKPANPVPP-----------------------KPPIDP--KKLIGIGILP
KDQVFSLFDERTWDEVSEVLKRFLAA--PTFEDFIKVAETLYPHVNEDLFLFGLSVAIVH
RPDANGVHVPRVHDVYPDKFLTNDVLINIRKQI-------IKGH---KNPVVKDTH--YY
HNDFDPFRRVDYFTEDPGMNSHHYHWHVLHPSIWHQ-GV-----G-E-KAKLGELFYCMH
RQMVARFDSELLSVHLPRIPSLDDWNKKVKIGYAPHLTIQRTGYTYMNRPENLEIEDLP-
-ELTKGELMQWKNRIMEAIARHNITLKGGPG---KKLRVSPE----------TGIDVFGH
LIAATKNSSNRQYYGNLHSYAHVIAARIADSDGEHKEDNGAMYDVATSARDPLFYSWHKF
IDKIFTEYQNTLTPYTPYQLT--WPDVVIDGIHIENLKT---------------------
------------------------------HEENMIHTYYTSSTFRLSKGFDYTKDS-EA
```

KVIVEHTDHDDFVYVIDIDNNARVEKTAVLRIFLAPKYDERGHPLTLKEQRVMMIELDKF
KAPLKPGHNVV-RRCSNESSVTMPVDHIYGDITQ-----------TVDEDHC----HCGW
PEYLLVPQGDYDGMHYELFVMATNYDEDKDQNA------------DMSC--KC---HAHS
YCGNILGEYLDKRPLGYPFDRKIKAA--GWEEF--K--TQNMCDTDVVIKFSGKT-----
--------------KAQ-----------
>CanHcB1
M-KL------CILAVAILVSAAL--------------------ADKQC-PP-PTNVEAKQ
QLIDNIVSYINKPANPVPP-----------------------IPPTDP--SKMKGIGKLP
KDQVFSLFDERCWPEVTVVLKKMMAA--ATFEDFVKVAETIYPRVNEDLFLYALSVAIVH
RPDAQGVHVPRVHDVYPDKFLTNDILVRIKNLV-------IKGT---KNPVVNDTH--YF
HNHFDPYRRVDYFTEDLGMNSHHYHWHVLHPSIWNI-SM-----G-M-KDRVGELFYWMH
RQMVARFDSELLSNHLPRIAPLDDWNEKVKIGYAPHLTIQRTGYTYMNRPENLELEDLP-
-ELTKGEMLQWKNRILDAIHRHNIT--KLDG---TKLKMTEE----------DGIDVLGH
LIASTVNSVNRRYYGNLHSYAHVLAARMADTDGEHLEDNGAMYDVATSARDPLFYSWHKF
IDKLFTEYQMTLKPYSEYELT--WPEVVIDAVHVENCRT---------------------
-----------------------------NEQNHIDTYYTTSILRLTKGFDYTKDT-EA
KVMVKHMDHDDFTYVIDVDNNAKVEKTAVFRVFLAPKYDERGHPLTLKEQRVMMIEMDKF
KKVLKPGHNVV-RRFSNESSVTMDTDHIFGDIKT-----------TEDEDHC----HCGW
PAYMLVPQGDHDGVHFELFVMATDSEKDKVATA------------EVQC--HC--KEAFS
YCGTIYGDNPDGKPMGFPFDRKMKAK--GWEEF--K--TCNMQDADITIQFTQDV-----
--------------KSH-----------
>ProHcB1
M-RL------LVLALACLAAVAYA-------------------GDV-C-QP-ATNVESKQ
GLINNLLTYINKPVNPSPP-----------------------VPQVDS--SKLKGIGLLP
KRKVFSVFDERVWPETERILEKFLTA--PTFEDFIKVAETIYPRVNEDLFLYCLSVAILH
RPDATGVHVPKVHMVYPDKFIHRDVIMRLKNKV-------MAGQ---KNVIINGTL--TH
NNYLDPYRRVEYFTEDIGMNSHHYHWHVLHPIVWSS-KL-----GNP-KDRPGEFFYWLH
RQMVARFDSELLSEDIPRIEPFDNWNEPIMIGYAPHLTVQRTGYEYMFRPEGLVLDNLP-
-ELTKDEMYQWRMRILEGVHRGRLT--LDNN---TKHELHGEDHHDNDEDEIHAIDTLAS
VVVSSRTSLNKEYYGNLHNYAHVIASKIADSHGQHMEDYGVMYDATTSARDPLFYSWHKF
VDSIFTEYQHTLEPYTHYELT--WPDVSLEELYVLDDEE---------------------
-----------------------------NEVNNLRTFYDTIPMKLYKGFDFTRDT-EP
TLLIKHLDHDDFTYHIKAKNHAKTPKKAVIRIYLAPKYDEHYHAFEPNEQRLLVIELDKF
STALKPGVNNP-ERHSNESTVIMNKNFIYGDGRLA---------KIHDHDHC----WCGW
AENLLIPKGTYEGMSFYLFVMATDYESEEIPDA-----------LNNPC--LC--KDAHS
YCGTIFGKYPGKRPFGFPFDREISEQ--GWEEF--E--TQNMISEEITIHFTCEGEH---
--------------HRH-----------
>SmuHcD
M-GV-----WSLLVFVVLFAFARSHHH----------------HES-CSKVYAQDYQLKQ
QEIQDLVDLINKPIRPDYQ-----DS----------------RYVVEE--RDLKGLGRLP
HDEVFSLFDERNWDETLQVIHLLTHA--PTYSKFLETARVVLPRVNEDLFLYALSVTLTL
RPDTRGVHVPRVLDSYPDKFLSRFVIVRLKELT-------RNGQYRNQVPIVDATQ--VR
SQSRDPNHDLDYFLRDPALNAHHYHWHVQHSPLLNE-KF------GH-SDRQGEYFFWMH
KQMVSRYDTALLSAGLRRVSPFENWNEPIQIGNAPNLEIDKTGYRYAYRPPGMVLENLP-
-NLPKNRMNEWRNRLVASIHKGYLV--AENG---SRVVLDGE----------TGIDYLGN
TIEANKQSVNSRLYGNLHGYAHIIAGKIMSPKGAPGERNGAMYDVATSAQDPLFYSWHRY
IDNILQEYKE-LHSYSRKELT--WDDVEVNQVQVKTIAD---------------------
-----------------------------GEQNTVRTYWQTSLYSIGKGFTFTQNS-RA
LVKFRHLNHEPYYYEIHVNNKAKSQRNAAVRIFGAVTEDERGEQLPLNSQRHLIIELDKF
VYPLQPGDNKI-VRYANQSSVTASEDSFFKPINQ-----------LPPESSC----HCGW
PQYLLLPKGRYEGLPFRVYVVVTDWDQDKVS-D------------EEQC--LC--TDATS
YCGTPNQINPDGRSLGFPFDKRIKER--NFQFF--R--TPNMGSTDINIRFTGKYLPSVI
GKQH----------KQY-----------
>SbaHcD
M-GV-----WSAFVLLAVFAIVKGHHH----------------YEK-CSKVYTNEIQSKQ

QEIQDLVDIINKPVRPEYQ-----NS----------------RNVVEE--KKLIGLGRLP
RDEIFSLFDERNWDEAIQIIRLLTEA--PSFEHFLATARVILPRVNEDLFLYSLSVALTL
RPDTRGVRSPRVLDVYPDKFLPRDVIVRLKEAT-------RWGQYRDKVPIVDGTV--GN
SNDPDPNHELDYFLRDPGLNAHHYHWHVQHSPLLDN-SM------QH-SDRQGDHFFWMH
KQMVARFDNSRLSAYLPRVIPFENWNEPIQVGNAPNLVIDKTGYRYAYRPPGIVLHDLP-
-KLPRNKMIGWRNQLVTSIHKGYLV--AANN---TRVNLDVD----------NGIDYLGN
AIEANRQSVNPELYGNLHGYAHVIAGRVVAPKGAPGEKNGAMYDVATSAQDSLFYSWHKY
IDNILQEYKE-LHSYKREELL--WDEVEVNQVRVKTVSS---------------------
------------------------------DVKDTVRTYWQNSYYPIGQGFTFTQNS-RA
LVKVRHLNHEPYYYVISLRNKANAIRNAAVRIFGAVTQDERGQSLPLNSQRHLIIELDKF
VVPLKPGENII-TRQANESSVTIPEDYIYNPVNE-----------VRPDSAC----HCGW
PQYLNLPKGRYEGLPFRVYVVVSNWDQDRVT-D------------EVSC--LC--SDAIS
YCGAPGQINPDRHSLGYPFDRRINQR--TFEAF--K--TPNMGSTDINIKFTGQYLPSSN
VNRF----------QHYSSNVYS---VL
>SweHcD
M-DT-----RVLRLTLALVALSGVLAE-------------HHHDEH-CKNAYVADYKLKQ
REVQHLVDSINKPVYPDFK-----DT----------------RGIIDE--RKLKGLGTLP
RREVFSLFDERNWAEAEQVVELLLEP--TTFREFIQLADIIRHRVNEDLFLYAFSVAIAH
RPDTQGVQVPRVQDSYPDKFLRKEIIHKIKEVM-------NEGQYLDKIPVIDATE--VS
ENHLDTNQELLYFLEDIGMNSHHHHWHVIHPAIWLP-KH-----GGL-KDRKGELFFYMH
KQMVARYDTERLSNNLARVRPFENWNEPIEEAYSPHLIVDKIGYKYAYRPHGITVQNLP-
-NLPKSKMVEWKNRILASIRKGSVV--ASNH---TYISLNDN----------HGIDILGD
IVESSLLSVNRVLYGNLHCYAHVIAGKVTDPHNTYEEKNGAMYDVATSARDPLFYSWHKF
IDNIFQEHKETLHPYTKDELN--FPEVQVDNLRVTVENG---------------------
------------------------------TYENIVRTYWQNSLYHIAKGFTFTPTG-PV
LVKVKHINHETFYYNLEITNNALTEKHGVVRIFGAVIKDERGHTLNLNDQRHLAIELDKF
TVTLQPGKNNI-RQPCYESHVTAKWDVFFSDVNS-----------QKPEEGC----NCGW
PDYMLIPKGKYEGLKFRVFAVVTDYDQDKTS-D------------HDPC--LC--GDAVA
YCGAHNQQYPDKKPMGFPFDRHIDDR--TFDQF--H--TPNMIATDVSIKFTGEFLPPK-
--------------GDI-----------
>ScoHcD
M-DT-----RVLRLTLALVALSGVLAD----------------SES-CSSAIVNDYKLKQ
KEIQHLVDTINKPVYPDFK-----DT----------------RGIIDE--SKLKGLGTLP
RREVFSLFDERNWAEAAKVVELLLEP--KTFREFIHLADIIHHRVNEDLFLYALSVAIAH
RPDCQGVQVPRVLDIYPDKFLRKEVIHKIKEVS-------NEGAYLDKVPVIDATE--VS
DNHLDPNQELLYFLEDLGMNSHHHHWHVIHPAIWLP-KH-----GGV-KDRKGELFFYMH
KQMVARYDTERLSNDLPRVRPFENWNDPIDEGYSPHLIIDKTGYKYAYRPQGVIVHDLP-
-NLPKTKMFEWKNRIMVGIRKGSLI--SANK---TQVPLNND----------HGIDLLGD
VVESSLLSVNRVFYGNLHCYAHVIAGKVTDPQSTYGEKNGAMYDVATSARDPLFYSWHKF
IDNIFQEHKETLQPYNKDELN--FPDVQVDSLRINVANG---------------------
------------------------------TYENIVRTYWQNSLFKIAKGFTFTTEG-SV
LVKVKHLNHETFYYNLEVTNNALEEKHGVVRIFGAVINDERGHPYILNDQRHLVIELDKF
TVNLKPGKNSV-RQPCYNSAVTAKYDVFYGDVES-----------QKPQEGC----NCGW
PDYMLLPKGKYEGLRFRVFAIVTNHDEDKVS-D------------QETC--LC--GDAVA
YCGAHNQKYPDKKPMGFPFDRRIDER--TFEHF--H--TPNMIATDVIIKFTGEFLPPK-
--------------GDI-----------
>SguHcD
M-DT-----RVLRLTLVLVALSGVLSL----------------HKQ-CEHHVVNDHKLKQ
REIQHLVDTINKPVYPDFK-----DT----------------RGIVDE--EKLKGLGTLP
RREIFSLFDERNWAEAARVVELLLEP--KTFKDFLQLANIIRHRVNEDLFLYALSVAVAH
RTDTQGVHVPRVQDSYPDKFLRKEVIHKIKEVM-------NEGAFLDKIPVVDATE--VS
ENHLDPNQELLYFLEDLGMNSHHHHWHVIHPAIWLP-KH-----GGL-KDRKGELFFYMH
KQMVARYDTERLSNDLPRVRPFENWHEPIEAGYAPHLVIDKTGYKYAYRPQGVIVHDLP-
-NLPKNKMFEWKNRILSSIRKGYLT--ASNY---SQIPLNDE----------HGIDLLGD
AVESSLLSVNRVFYGNVHCYAHVIAGKVTDPSGTYGEKNGVMYDVATSARDPLFYAWHKF

IDNIFQEHKETLHPYTKDQLN--FPDVQVDSFSVTVVNG---------------------
------------------------------SNENIVRTYWQNSMFKISKGFTYTPTG-PV
LVKVKHLNHETYYYNLEITNNALSDKHAVVRLFGSVINDEHGHSLNLNDQRHLVIELDKF
TATLKPGKNKI-KKGCYESHVTAPWETYFSNVNS-----------QSPEEGC----HCGW
PDYMLIPKGKYEGLPFRVFAVVTDYDEDRAS-E------------QEPC--LC--SDAVS
YCGAHNQIYPDKKPMGFPFDRRIDDR--TFELF--H--THNMIATDIKIRFTGEYLPPK-
--------------GDI----------
>NadHcD
M-RT-------WTFLIFLWGIAHAHHHLGQQWQGQRYHYQQQEGVHGS-WSVAQGYELKQ
QEIQSLVDLINKPVRPEYE-----YP----------------DDVVDE--DDLHRLGHLP
RDEVFSLFDERNAQEVIDTIRILYEA--PTYDEFLSRARVIRQRVNEDLFLYGLSVAITI
RNDTRGIHVPRVHDIYPDKFLTRDVIQRIKEAT-------RRGHYRDVVPVIDATE-PEA
YNSADPSQQLAYFLNDVARNSHHYYWHVLHPAVRGE-EL-----GGA-SDRQGEIFYWTH
KQMVARYDTELLSNQLPRATPFENWNEPIEIGYSPNLAIDKTGYQYAYRPSGVVLKDLR-
-NLPRNKMIQWRNNLVDSIHEGYLL--SENG---THIKLDNN----------EAINYIAS
ALEASRVSVNRRLYGNLHNYAHVIASKVTTPSEARWERNGVMYDVATSARDPLFYSWHKY
IDNILQEHKEQC-SYSREERS--WDDVEVNSVQVVAYGD-HPEDHQGHHGHGGQHWHHGQ
QWHQGGRYGNIRGGHYWNQQNAQTQKNERQSQPNVVRTYWQNSVYEIGKGFTYTPKS-KA
LVKLHHLNHEPYNYSISVTNNAGSQREVVVRIFGALTEDERGHTFDLNEQRHIVLELDKF
RAVLKPGQNKI-ERSSLDSSVTSHGRSFYEPVER-----------LYPEDAC----QCGF
PNYLLLPRGQPEGTNFQIYVVLTNAEEDIVQEQHHHEHQQQQQHQYQDD--IC--WKNIN
YCGSPWQRYPDRRPLGYPFDKRIDER--NFDSW--R--TSNMGSTNITIYFTGEYMEPSY
REYEWA----SIHLRRQ----------
>ScoHcC
M-GA---WKVWTFFAIALVVAVKAYDE----------------EAK-C-MSHDEDSQVKE
RQILHIVDSINKPISPDFR-----AP----------------RGVIDE--HKLRGLGTLK
KREIFSLFDERNWDEASKVVRLLLDA--KDFDDFIDVAEVIRLRVNEELFLYAFSVAVMH
RGDTQGLQVPRIHDIFPDKFLKEDVIHRLLELS-------NRGEHYDRIPIIDATQ--IS
HNYLDPNSELEYFLEDLGLNSHHHHWHVIHPAIWVS-EL-----GNE-KDRKGEFFYWMH
HQMLARYEAERMSNGLARTRTFQNWNDPIDEGYAPHISIMKTGYTYAYRPPGYTLRDLP-
-NLPKNKMVEWAKRVLYSIHSGIFH--FSNG---TDAHLDTE----------HGIDELGN
IVESSLTSLNRDYYGNLHCYAHVIAGRIADPEGKYGEDNGVMYDVATSARDPLFYRWHKY
IDNIFQEYKNTLPPYTTEELTPQNSEFRVQGISVVGET----------------------
------------------------------SARDTVHTYWQHSLLKVGQGFEFTKHT-PA
YVKVKHLQHESFTYVIDVENR-GRTRTGFFRIFAAPKYNELGQKWHINDQRLIMVEMDKF
IEKLYPGKNTI-ERHSEDSTVTMSSASIFSDISS-----------EQSEDHC----SCGW
PDYLLVPKGNFEGFPMEVFVIVTDYEEDKVEGP------------DEGC--AC--HDALT
YCGGIDYHFPDKRAMGFPFDRPIKQR--NFNAF--K--TKNMGKVTVDVKFTGETIAPED
FH------------NQH----------
>SguHcC
M-GA---WKIWTCVALALAVTVSAYVE----------------EER-C-DDVSDDTKLKQ
RQILHLVDSINKPIAPDFR-----AP----------------RGVIDE--HKLKGLGTLR
KREIFSLFDERNWEEASKVVQLLLEA--EDFEKFIDIAEVIRLRVNEELFLYAFSVAVIH
RGDTQGLQVPRIHDVFPDKFLKEDVIHSLQELS-------NRGEYYDRIPIVDATQ--IS
HNYLDANSELDYFLEDLGLNSHHHHWHVIHPAIWVP-EL-----GST-KDRKGEFFYWMH
HQMLARYEAERMSNGLARTRTFQNWNDPIDEGYAPHISIVKTGYTYAYRPPGYTLRDLP-
-NLPKNKMVEWAKRVLYSIHSGIFH--HPNG---SDVNLDVN----------SGIDVMGN
LIESSLTSMNRDYYGNLHCYAHVIAGRIADPEGKYGEDNGVMYDVATSARDPLFYRWHKF
IDNIFQEYKNTLSPYTHDELIPHASEFRIQGVSVVGET----------------------
------------------------------SERDTIHTYWQTSLLKVGQGFEFTKHT-PA
FVKVKHLQHEDFNYVIDVENR-GKTRTGFFRVFAAPKYNELGEKWHINDQRLIMIEMDKF
IHKLYPGKNTV-ERDIFDSTVAMSSANIFSDVTS-----------GQSEDHC----SCGW
PDYLLVPKGNFEGFPMEVFVIVTDYDEDKVKSQ------------QDSC--PC--HDALT
YCGGADYQYPDKRAMGFPFDRPIKER--TFDAF--K--TKNMGSVTVKVKFTGEAISPED
YH------------EEH----------

```
>SweHcC
M-GA---WKIWTSIAVALVVVANAYDE----------------ESI-C-TSHSEDASVKQ
RQILHLVDSINKPIAPDFK-----AP----------------RGVIDE--HKLRGLGTLK
KREIFSLFDERNWDEAAKVVQLLLDA--NDFEEFIDIADVIRLRVNEELFLYSFSVAVMH
RGDTQGLQVPRIHDVFPDKFLKEDVIHQLSELS-------NRGEHYDRIPIVDSGH--IT
HNYFDPNSELDYFLEDLGLNSHHHHWHVIHPAIWVP-EL-----GSE-KDRKGEFFYWMH
HQMLTRYEAERMSNGLPRTRTFQNWNDPIEEGYAPHISIMKTGYTYAYRPPGYTLRDLP-
-NLPKNKMVEWAKRILTSIHSGYFH--FENG---TKARFDVE----------HGIDHLGN
LIESSLTSINRDYYGNLHCYAHVIAGRIADPEGKYGEDNGVMYDVATSARDPLFYRWHKY
IDNIFQEYKNTLTPYTAEELTPHTSEFRVAGVSVHSGT----------------------
------------------------------SERDTIYTFWQDSLLKVGQGFEFTQHT-PV
FVKVKHLQHDTFDYIIDVENR-GKTRTGFVRIFAAPKYNEHGEKWNINEQRLIMIEMDKF
THKLYSGKNTI-ERSSSESTVTMNSAGIFGDVSS-----------EHAEDHC----SCGW
PEYLLVPKGNFEGFPMEIFVVITDYEEDKVTSH------------DEGC--PC--HDALT
YCGGIDYKVPDKRAMGFPFDRPISHR--FFDDF--K--TKNMAKTTVHIKFTGETIAPED
VH------------DEH-----------
>PanHcC
M-KVDSGFTLFFFLLAVSGLAVQAIIK----------------DTH-C-AQ-ATNTKLKQ
LQITQIISKINKPVPAEIR-----SS----------------ENIIEK--SRLRTLGQLP
RREVFSLFDARYWNETSQVLDLLIEA--KTFDDFIKRSEVIQPLVNEELFFYAFSVAFLH
RQDTHGLHVPRVHYVFPDKFLKTEVIQKMKQLT-------YNAERYGKSAIVEGNDLSHI
HNYYDTHDELSYFTEDVGMNAHHYHWHVVNPAVWKN-KY-----GNY-SDRKGELFYYMH
SQMVARYDIERLSNGIPRTVPFQSFNDEIEEGYNPHLTMAKTQYHYAYRPPHFTLQDLP-
-TLPKSRLQEWSNRLFHAIHTGQLT--RPNG---RKLALNNE----------HGIDYLAN
TIEANYDSVNYKLYGNLHCYAHVISAKITDPTTAYNEDYGVMYDVATSARDPLFYRWHKF
INKFFSEHKKMLKPYTKDELD--FPGVKVEKVSVQTST----------------------
------------------------------SEPNLIRTFWQDAHLKVREGFLFTRQS-PA
YVKLTHLDHETFTYRIDVNN-GGSAHEAVVRIFLAPVYDEFEHRFDIKHQKSLMIQMDKF
VTKLTPGKNTI-VRSSLNSTVTMEANSIFGAKRP-----------SKTIDNC----RCGW
PDYLLVPKGNYEGMKFQLFVMVGDWAKDENTES------------RGNC--FC--KDSLT
YCGGIDSKYRDTKPFGFPYDRKIKAE--SWQDW--E--TDNIAHTDITVKFVGDDLHDTH
PEEF----------SDD-----------
>PseHcC
M-KMDSGFTLSLLLVLVSSFAVQALVK----------------ETH-C-AK-PTNTKLKQ
LQILQVVSSINKPVPAEIR-----DS----------------TNIIDK--SKLRNLGQLP
RREVFSLFDARYWNETSQVLELLLEA--KTFDDFIKRSEVIKPLVNEELFFYAFSVAFVH
RKDTQGLSVPRVHMVFPDKFLKTPVIQKLKQLT-------YNAERYGKSPIVEGNDFSHV
HNYLDTHDELSYFTEDLGMNSHHYHWHVVNPAIWKT-KY-----GNY-SERKGELFYWMH
SQMVARYDIERLSNNLPRVTPFQSFDDEIDEGYNPHLTMAKTQYHYAYRPPHFVLQDLP-
-TLPKSKLQQWSNSLFHAIHTKQLT--RPNR---SKVSLNNS----------RGIDHLAN
TIEANYDSVNYQTYGNLHCYAHVIAAKITDPTTAYHEDNGVMYDVATSARDPLFYRWHKF
LNKFFTEHKKTLNMYSDDDLK--FPDVKVDKVSVQNQG----------------------
------------------------------SEPNLIRTYWQDAHLKIREGFLFTRQS-SA
YVKVTHLDHENFNYRIDVTN-TGGPHEAVVRIFLAPVYDEFERPLDIKHQRPLMIQMDKF
LAKLTPGKNTI-VRSSQNSTVTMDSRSIFDSDYH-----------SKTNDNC----RCGW
PDYLLVPKGNYEGMKFQLFVMLSDWAKDEDTHT------------RGNC--FC--KDSLA
YCGGLDAKHRDVRAMGFPYDRRIKAE--SWDEW--E--TDNIAHTTITIKFVGDELHETH
PEHF-----------ED-----------
>AmaHcC
M-RL-------LFGLAALLLLVTLV--------------VAKREDL-C-EY-PKDAKLKQ
RQIINIISTINKPISPIYR-----ES----------------RDVLTE--YQLQGLGILP
KTEVFSLFDERYWNETIHVLELLFEA--HDFDDFIKRSEVIRLRVNEELFFYAFNVAVLH
RSDTQGLQLPRIHKVFPDKFLKESVMRKMIETN-------QKAKYIGKSPIIDTAG--DL
YNHLDMNSELNYFTEDVGLNSHHYHWHVLNPAIWLE-EF-----GGK-KDRKGELFYWMH
QQMVARYDIERMCNHLPRVKALWNLNEEIEEGYEPHLTMVKTQYHYAFRPPHSTLHDLP-
```

-DFPKSKMFEWARRILDGIHKGFFI--HKNH---TMVKLDEK----------NGINLIGN
AIEANLDSVNCHLYGNLHCFAHMLAASVTDPLGTFGETHGAMYDVATSARDPLFYRWHKY
INHFFEQHKKMLTPYTHDDLE--WSDVHVNGISVQGRG---------------------
-----------------------------SGPNEIRTYWQRTLSQIPKGFEYTKEY-PA
KVAYTHLQHENFDYVIEIENRARTAKLGLVRIFIAPVYNELGRKFTINEQRLLVIQMDKF
KVRLSPGRNTI-RRNADNSTVTMTSKNIFAPITQ-----------LKDEDHC----RCGW
PDYLLVPRGDFNGKKFRLFVIVTDFEKDFVP-V------------SGSC--MC--RDGLG
YCGGINTKYPDRRAMGFPFDRKIHHF--NWDEF--E--TDNMARTKISIRFTNEFMPPRD
YDDH----------QFR-----------
>CleHcC
M-RL-----LVCTLVAALVVVATGADR----------RDFPKKTDF-C-GE-PKDAALKQ
RIIINLISTINKPVSPVYR-----DS----------------HGIIDD--YKLMELGVLP
RDQIFSLFDERNWNETITVLDLMFGA--KTFSDFIKVSEVIRFHVNEELFFYAFTVAVLH
RKDTKGLQVPRVHKVFPDKFLKEAVIQKLRETS-------KKAKYMGRSPVVLGSV--DL
FNHQDINTELNYYTEDVGLNAHHYHWHTMNSPLWKD-SF-----GGH-KDRKGEFFYYMH
QQMLARYDVERLSHHLPRVKPFQNWNSKIKEGFDPHLTMVKTQYHYAYRPPGYTLKGLR-
-DLPKSKLSVWEERLYYAIHRGFVL--DKHN---HKIYLNGT----------DGIDILAN
AIEANLDSVNCHLYGNIHCYAHVIAAKITDPLEAFGETAGAMYDVATSARDPLFYRWHKY
IGNFFLEHKNMLTPYNKTDLN--WDDVTVKGISVHGRG---------------------
-----------------------------SAHNVIHTYNQHTLMRIPKGFEFTQDF-SA
IVKLQHLQHESFEYVIDVVSKAATPKLGLVRIFLAPVENEMGKRFTINEQRQLMIKLDTF
STQLHPGKNII-RRSSENSSVAMTSHSLFDDFED-----------LKDEDHC----RCGW
PDYLLLPRGDFDGMKFELFVTITNFKRLEST--------------TGNC--MC--LDGLA
YCGGVDVPYPDKKPLGFPYDRKIKNL--TWDEF--E--TDNMAHTPITIQFTGEFLPPRD
F-------------FHH-----------
>CanHcC
M-RL-----LLFSFVVSLTVVSTWG--------------LIRTKHY-C-DL-PNDIILKQ
RQIIHIISSINKPITPDFR-----ET----------------EGVLEE--YQLRGLGHLP
KNEIFSLFDSRNWNETIKVIKLLLKA--PTFDDFVKRSEVIHLRVNEELFYYAFSVAVLH
RQDTQGVHLPPAYKVFPDKFFKDHVIHKLTEVT-------RKSKYLGKTPIVDGTE-EDP
YNYLDPNHALEYFTEDVGMNSHHYQWHTINPAIWLN-KF-----GDY-KDRKGELFYWMH
QQMIARYDAERLSNYLPRIKPFEDWDEKIEDAYNPHLTMAKTQYQYAYRPKNYVLRDLP-
-ELPKSKLMTWTLSLANAIQRDRLL--ASNG---TIVPLNCS----------SGIDEVGN
AVEANFDSVNMKLYGNLHCYAHVIAGKITDPKGSYGQENGVMYDVATSARDPLFYRWHKF
INSLFVKHKKMLTPYTKDELE--WTNVEVRSVSVRGHG---------------------
-----------------------------SRSNKIRTYWQNTLVKINQGFVFTRES-PA
YVKLHHLQHENFDFIIEAVNRASSPKYGTVRIFMAPVYSELDRKFTVNEQRLLMLEMDKF
VTKLAPGKNTI-TRNSKNSTVTLSSENLYAPSMA-----------LRLNDHC----KCGW
PDYMMIPKGTHSGMEFQLFVTITDYEEDKVK-S------------QGSC--FC--KDAFS
YCGGIDAKFPDKRAMGFPFDRKIKAY--GWDDF--E--TDNMVHTTVIVKFTGETLAPQD
F-------------YHL-----------
>ProHcC
M-RL------FVFLVAALAIAATEA--------------LVKKDDR-C-EV-ADDLILKQ
EIVIQLISTINKPFSPRYR-----DP----------------KGILEP--HQLKGLGKLP
RNEIFSLFDQRNWDETIHVIELLLDA--VDFDDFIERSEVLLLRVNPELFYYSFAVALLH
RDDTRGIHVPRVQYVFPDKFLKEPVIEKLKHLT-------SIPA-GNQPPIVDFHE-GDE
GSTLDPNWAVTHFTEDIGLNSHHYHWHTVNPAVWQD-KF------GE-KDRKGELFAYMH
LQMINRMDAEYLSEHRPRIKPLNNWDDEIEEGYDPHLAIAKTRYHYAYRPPHYVMKDLP-
-DLPKTKMAEWSVRLFNCIHRGRLI--AANG---TEVPLDPE----------HGIDILAN
AVESNYDSVNPDFYGNIHSYAHVLAARITDPSGAYGEDSGVMADTATAARDPLFYRWHKF
IHHFFREHKEMLPPYTREELN--FDDVIVKGVTVQGEG---------------------
-----------------------------SKPNLINTHFQNTLFKVHQGFVFTKNS-KA
YVRYPHIQHEDFEYIIDVENK-GVSKKAVVRIFIAPFYDELGRKSTPNQVRRSVIEMDKF
SVKLTSGKNTI-RRSCANSTVTAPARSIFGSPKD-----------LLKEDHC----RCGW
PDYLLVPTGTREGMKFHVFVILTDYDEDKVTDG------------HHQC--YC--KAAVS

YCGGIEAKYPDRRAMGCPFDRKVKAC--DWQQF--K--TENMAAGKFIIKWNDDFLPPQD
FHH-----------RRY-----------
>PanHcBII
M-KS-----VLLIVIAVCVSAIN--------------------AA--C-PAPTGDVKLKQ
SQIQKIVEHINRPYVAETE-----------------------LLTGDP--AKFKGLGNLH
HEQIFSLFDERQWPEAIKVLELLTGA--PTFEEFLKVAEAIRQKVNEDLFFFAFTTAVVH
RPDTQGLQVPRIQDVFPDKFFTQEVIAQIKEAS-------HRGE---KNPIVNDTC--HY
GNILNVNNRLTYFTEDMGMNAHHFHWHAVNPAIWLD-KF-----GGV-KDRRGEAFYWMH
RQMLARYDAERLSNNLPRTTPFEEWDHPIHEGYAPHLTVDRTGYTYSFRPEDLTLHNLP-
-ELTKNQMLLWRNRIWDTIHSGILY--AKNQ---TKIRLLDD---------PQGTDLIGH
ILESSLQSVNRPFYGNLHCNAHVIASEITDPDGKYEQYHGAMYDVITSARDPLFYRWHKF
IDNIFHDYKNTLPEYKSTEVA--WGEVQIESAEIKGDKS---------------------
------------------------------EDKNLITTFWDHHPFKIGKFFSFTAQN-TA
EVRISHLQHEGFTYKFDLVNNAGDSKEAFIRVYLAPKYDETGKKFTADQLRPLLIETDKF
LVKLKPGKNSI-TRHSSESSVTVPDKQIFGLQDT-----------TESKDQC----RCGW
PNYLLVPKGKREGMKFRLIVVATNWADDKLH-D------------DTSC--MC--KGSPA
YCRLLYNQVPDRRPLGYPFDRVIGTR--KLEDIHQK--VQNLFATDVTIKFTGEAL----
----------------H-----------
>PseHcBII
M-KS------LFILLAICVAAIH--------------------AA--C-PAPTGDVKLKQ
SQIQKIVEHINRPYVAETE-----------------------LLTQDP--SKLKGIGKLP
HHKVFSLFDERQWPEVVKVLELMTSA--STFEDFLKVSEAIRQRVNEDLFFFAFTAAVVH
RPDTQGVHVPRIQDVYPDKFFTQEVITQIKEAI-------HRGE---KNPIVNDTC--HY
GNTLDINNRLTYFTEDLGMNAHHFHWHAVNPSIWLD-KF-----GGV-KDRRGEAFYWMH
RQMLARYDAERLSNFLPRTTPFEDWDHPIHEGYAPHLTVDRTGYTYAFRPEDLVLHNLP-
-ELTKNQMLLWRNRIWDTIHSKVLY--AANG---TKVELRED---------PEGTDVLGH
LIESSLQTQNRLFYGNLHCNAHVIASRITDPDRKYEQYNGVMYDVTTSARDPLFYRWHKF
IDNIFHDYKNTLDEYKPTEVS--WNDVRVESAEIKGDKS---------------------
------------------------------EAKNLITTFWDTHPFKIGKFFSFTAQS-TA
EVRVSHLQHEGFTYKFDLVNNAGDSKEAFIRVYLAPKYDETGKEFTADQIRPLLIETDKF
LVKLKPGKNSI-TRHSSESSVTVPDKQIFGSLDT-----------TESKDQC----RCGW
PNYLLVPKGKCGGMEFKLVVVATNWADDKLH-D------------DTSC--MC--KGSPA
YCRLLYNQVPDRRPLGYPFDRRISTL--KLEDIHTS--VQNLFATDVTIKFTGGSMA---
----------------H-----------
>AmaHcBII
M-RL------AIAILALCLTQAL--------------------AS--C-PAPTGDLKAKQ
AQIQNIIEHVNRPYLSDDD-----------------------HAKTD---MDLPGLGVLP
NHEVFSLFDERQWPEVTKVLKMMMGA--KDFEDFLRVSDAIRHKINDDMFMFAFTAAIVH
RPDTQGLNAPRVQDVYPDKFLTHDVIIKIKEAI-------SRGV---KNPIVNATH--DF
WDIRDQNNKMSYFTEDLGMNANHYHWHVVNPAIWTN-EL-----GGE-KDRRGEAFYWMH
RQMVARYDAEKLSNKLPRTSSYENWDSPIRSGYAPHLTIDNTGYNYMFRPDDVVMPNLP-
-ELTKNQMRLWRSRILDSIHKGYVY--ARNG---TKIMLDHD---------PFGIDVVGH
MVESGRLSVNRPLYGNLHCYAHVLAGRTVDPYGKYEQDNGAMYDVATSARDPMFYSWHKF
IDNIFQEYKDTFTGYTPEEVT--WKDVEIESVSVKGEKS---------------------
------------------------------EQSNRLTTFWDTNLFKIGKYFSFTGEN-TA
EVRVKHLQHEGFNYKFEISNNGGEAKEAFVHVYLAPKYNEKRKPFTPDEQRQLMIQLDMF
ITKLKPGKNSI-TRHSSESSVTIADKQIFAPADT-----------LDGKDQC----KCGW
PNYMLLPRGRTEGMVFQLIVAVTNWGEDKQT-D------------DTSC--MC--KGSPS
YCRFTLDKVPNRRPLGYPFDRKITST--SWDEI--K--VQNLYTTDVVIKFTGDNLTP--
---------------AQ-----------
>CleHcBII
M-KL------TIAILALCLAQAL--------------------CDPLC-PAPTGDLKAKQ
AKITDILEHINRPFEPDTH-----------------------KVNIND--ERLKGLGVLP
DHEVFSLFDARQVPEATRALKLLMGA--KTFDEFIDISETVRHVLNDDMFLFIFTSAIIH
RKDTQGLNAPRVQDIYPDKFLTQDVITKIKETI-------NRGE---KVAVVNATH--DY

```
WDTRDINNRLSYFTEDLGLNANHYQWHAVNPAIWKG-AY----MGGV-KERRGEAFYWMH
RQMLARYDAERLSNNLPRTKPFENWDSPIRQGYAPHLTIDRTGYVYMFRPDDVVLPNLP-
-ELTKNQMRLWKARILNCIHRGSVY--HRNG---TKLDIVHT---------EHGIDTIAS
LVEASFDSVNFNFYGNLHNYAHVLASRIVDPDGKYEQDNGAMYDIATSARDPLFYKWHKF
IDNIFQEYKDTLKTYSTEEVT--WSDVEIESVSVKGDKS---------------------
------------------------------DTPNKLTTFWDTDIVHIGKHLSFTGES-TA
DVRVKHLQHEGFNYKFDITNKGGEAKEAMVRVFLAPKYNEQRKPFKPNEQRQLVIELDKF
VTKLKPGKNPI-TRHSSDSSVTIADKQIFAPVDT-----------LDSKDQC----KCGW
PNYLLLPRGKPEGMVFQLFVAVTDWAHEKVS-D------------DISC--MC--KGSSS
YCRFLYGKIPDSRPLGYPFDRKITST--SWDEI--K--IQNLYSTEVVIKFTGDNLTP--
--------------PQA-----------
>CanHcBII
M-KL------LILAVALFAAQAY--------------------AK--C-PAPTGDLKLKQ
SQVQKIIEHVNRPYLPDSD-----------------------SSKLD---LSGRGLGYLK
KWQVFSLFDERQWYEAEKVLDLMMSA--KTFEEFLDMSDAIRHKVNPDLFLFAFTTAIVH
RPDTQGLNAPRVQDVYPDKFFTKDVIAHIKEAV-------NRGE---KNPVVNDSC--ES
WNIMSDENKLSYFTEDMGMNAHHYHWHTVNPAIWTE-KL-----GGM-KDRRGEAFYWMH
RQMVARYDAERLSNNMHRVEPFEDWEDDIDEGYASHLTIDNTGYHYMFRPDDMHLNDLP-
-ELSKNELKLWKSRILDSVHKGYVY--AKNQ---TKINIKED---------PFGIDVLAH
IIESSRQTINRPYYGNLHCYAHVIAARIADPDGKYMLDNGVMYDVATSARDPLFYKWHKF
IDNIFQQYKNTLPPYPNEEVN--WSEIKINSVEVEGEVT---------------------
------------------------------KTPNLLTTFWETDTFKIGKHYSYTAQS-TA
EIVVRHLDHEPFTYKFEVVNNAGDSKEAFVRVYLAPKYNEFGKPFKPNQLRQLVIEMDKF
HMKLKPGKNTV-TRLSSESSVAVPKKQIFGPVDT-----------LDNKDQC----KCGW
PDYLLLPRGKPEGMPFTLIVYMSNWAEDKIH-D------------DRSC--LC--KGAPS
YCRFLYEKVSDKRPIGYPFDRKVEST--TFDEIEAQ--IQNIYHQDITIKFKGNTLLH--
--------------RSP-----------
>ProHcBII
MMKL-----TQFIIFGLCLALAL--------------------ADKKC-QDATGDLKLKQ
SLIQKIVDHVNRPYLPDTE-----------------------APTYDS--PKMKYLGKLP
KTEVFSLFDERQWPEAEFVLKLMMKA--TDFDEFLRVSDAIRHRVNNDLFLYTFSVAITH
RPDTKGLMPPRVQDVYPDKFFTHDVIVKIREAA-------NRGE---KNIVVNDTH--DY
WNNLDLHHRLSYFTEDLGLNSHHYHWHVMNPALWSS-KL-----GEV-KERRAELFAWTH
KEMLARYDSERMSMDLPRTRPFEDWDHPIHTGYAPHLTIDRTGYIYSFRPDDMVLKNLP-
-ELTKHQLRLWRGRILDSIHKHYVY--ARNG---TKISIREE---------PEGTDIVAS
LVESSRESINRPYYGNLHSYAHVIAGQIADPDGKYEQDHGVMYDVAVSARDPLFYKWHKF
INNIFREYKETKTGYTPAEVT--WSGIDIESVKVQGEKT---------------------
-----------------------------TSVPNKITTFWDHSTFKLGKHYTFTGQS-TA
EINVQHLQHEDFSYDIELVNNAGGEKNALVRIFLAPKYDEQHKPLKPNDQRSLAIMLDQY
IAKIKPGKNTL-HRHSSESTVTVPDKYIFAPADTL----------DYKEQTC----RCGW
PNYLLVPRGKPEGMTFKLIVYISNYDEEKTD-Y------------DKSC--YC--PGSGS
YCGIVAGKLGVKTPLGYPLDRKIDGL--KWKEI--Q--GPNLYTSDVIIKFVGDNLTP--
---------------TC-----------
>AmaHcD
M-ML------KVALCLLVLFVANAYSD----------------KLK-C-RTAVPDFKLKQ
RMINHLVDFINKPIYPDFT-----SA----------------YGILTQ--KEEMGLGRLS
PDQPFSLFDERHWREAKKVIKLLLNA--RDFEEFVKIAELLHERVNDDLLLYGVSVVLLH
RNDTQGLHVPPIHKIYPDKFLRASVIKEMKELS-------KQARLLDETPLIDATE--DH
DNNLDPNHDLTYFLEDLGLNSHHYHWHVMYPALWK---------GDD-LDRKGELFYWMH
KQMLTRYEIARMCNGLERVLPFENWNEPIEEGYNPHLVIDKLGYRFTYRPPHMVLRNLP-
-NLSKVDLLQWKENIMYAIHRERVI--AENG---TVIPLNND----------HGIEILGD
IVEASALSVNKGTYGNIHNYAHVMAAKITDPYDQFGGNYGAMYDVATSARDPLFYSWHKF
IDNIFQEHKNMLPPYTKEQID--FPNVKINKLTIKGDNT---------------------
------------------------------TEDNIVHTYWQDSVVKVPKGFTFTGET-AM
YVKVRHLQHENFKYEIEVEN-GGPEKTAVVRIYLAPEENELHQKLRPNEIRQLNVELDKF
```

```
TVKLQPGKNVI-LRASDESTLTTPWSHIFKDRYK-----------SEPMDHC----HCGI
PDYMLLPKGSYSGMPFYVAVHIADWEEDRVD-Q------------VENC--HC--PDYVN
YCGDADRRYPDKRPLGFPFDRRVEG---MWDTF--A--TPNMIHQDIQIKFTGEVLAPKS
YYYPHH------K-MNV-----------
>CleHcD
M-KF------LLAAVVLALCLASGLAD----------------KVQ-C-ITAVPDFFLKQ
KQINHLVDFLNKPIFPEYT-----SA---------------RGTLTP--YELVGLGKLA
PDQPFSLFDERQWKETKKVIKYLLRA--KDFDEFLHVAELLHERVNDDLLLYGLSVVLLH
RNDTQGLHVPPIHKIYPDKFLKVPVIQKMKSLA-------KKYKYLDETPIVDGTD--EP
GSIDDPNHDLHYFTEDVGLNAHHYHWHVLYPALWK---------GDD-LDRKGEHFYWQH
KQLLARYEVERLANGLSRIRPFENWNLPIEDGYAPHLVIDKLNYRFTYRPPGVTLKNLP-
-TLSKDSMIEWKEALLNAVHLGNVL--LPNG---TKIPLNNE----------HGIEILGD
LVEASRLSKNKELYGNIHNYAHVMAAKVTDPYDRFGGNYGVMYDVATSARDPLFYSWHKF
IDNIFQEHKNMLTPYTKEEVE--FPGVKIDKLTIKADNT--------------------
-----------------------------TTDNVIHTFWQESTFKVPKGFTFTPTT-DM
YVKVRHLQHEVFKYELDVSN-TGALRNAVVRLFLAPIYDEMHRPFSSNDRRQLVIELDKF
TTKLQPGKNTI-IRDSSDSNLVTPWSHIFKDKYK-----------SQPMDHC----HCGV
PDYLLLPKGDFDGTEFYFFAIINDWDHDKVK-Q------------AENC--HC--PDSAN
YCGDADRLYPDTRAVGFPFDRKLRET--KFADY--S--TENFVRTPVTIKFTGEILPPKA
YYKDRP--------QHN-----------
>ProHcD
M-IK------GALCLVALLAIGTGWAV----------------ELQ-C-HDTVPDFKLKQ
RYINHLVDLINKPIYPGYT-----PI---------------QDAFDG--HELKHIGELS
PDKPFSLFDERTWKEAREVIDLLLDA--SDFEHFVERANLLHERVNDDLFLYCLSVVVLH
RNDTQGVHVPPVHKIYPDKFLKLDVINKMKRLA-------KKSRYFNEKPVIDATD--YH
ENHLEPNHDLMYFLEDIGMNSYHYDWHVMYSALWN---------GDD-ISRQGEMFVNMH
KKMVNRYDIERLANGMPRVKPFNAWNEPIEDGFNSHLLIDRIGYRYAFRPPHMILRNLR-
-KLSKNQLLVWEQRILNSVHRGIVI--AENG---SYVPLDDH----------DGIDVLAK
MVEASVKSINKGFYGNIHSYAHVLAGKITDPDDRFGGSNGVMFDVATSARDPLFYSWHKY
LDKIFQEHKKQLTPYTTEELT--FPSVKITRLLVHGENS--------------------
-----------------------------TEDNVLRTYWQESLLKVKKGFTFTGDM-PI
LVRVTHLQHEDFHYEIDVEN-GGAERRAVVRISLVPLYDETNHSLSMEEKRNLAIDLDKF
VVPLKSGVNKI-VRHSNESNLVTPWTHIFDDKYK-----------TDPLDHC----HCGW
PDYLMIPKGNYEGMHFGVAVVITDWDEEKVD-Q------------PENC--HC--SERTS
YCGDVDHLYPDTRAMGFPFDRKLAPE--VWENF--G--PDNIDRVTIKVKFTGKTLPPKN
IERLYE------KERRL-----------
>CanHcD
MIRT-------SVLVLALLAIGGGWAA----------------KLK-C-RDAVPDFHLKQ
RQLNAFVDTINKPIVTDFK-----ST---------------KGNIDV--YDLKYLGHLP
KNQPFSLFDARYYKEARQILKLLSDA--KDFDHFLHTAEILHGMVNEDMLYYCLSVVVLH
RNDTQGLQVPAVHRVYPDKFLKIDVIQKMKELV-------QDGDHLSEMPIIDTTE--ES
INYKNKHYDLNYFLEDIGMNSHHHQWHVLYPAIVE---------LDT-LERKGEMFYWMH
KQMLARYDAERFSNGKAPVKPFINWEDPIKDGYAPHLRIDKMGYRYAYRPPNMLLKDLK-
-DLDKEDMLTWGERLLKAADLGIAR--ASNG---TIIDLCDE----------HGIEIMGD
MVESSLTSINREYYGNLHSYAHVMAGKITDPKDVHSGHYGVMYDVPTSARDPLFYAWHKY
INRIFEAHKNRLTPYTREELD--FANVQVKSVKVMA-----------------------
-----------------------------NKENVIRTYWQESILKVPKGFEFTGQT-TI
GVKVRHLQHEPFNYVIEVEN-GGPPKMAVVRIYGLPNY--WGPHKTFNNMRKYQIELDIF
TTKLESGMNKI-VRAANESNLITPWNHVFKDYTK-----------SGPMDHC----SCGW
PEYMQIPKGTYAGMPFYVTAVVNDWDQEKVE-Q------------EDNC--HC--KDPVT
YCGDVDRHFPYKKALGFPFDRKAPVY--TFDEW--A--TPNMRYAKTKITFTGKTL----
---------------TH-----------
>ScoHcX
M-KYCTESLILILAVIGCISAAI--------------------NFK-C-PK-GTAEEKHQ
KEIYDLVQRINRPLIPQFK-----EPNFPT-SFLI-------KGKDPK--EFFQAIGHLP
```

```
KKEVFSLFDERHWDEAMTAYEYLYEA--ETLDDFIDIAKILYLHLNEDMFYYVFSFAVLY
RKDTRNVRLPQVHDVYPDKFLKTDIINKIKQAN-------YQGK---QHPVIDATK--EF
HDLRNPVSYLHYFLEDIGMNSHHYHWHVMNSALRKA-YP--TE-GEKKFYRKGELFYHMH
HQMLNRYELERLSNGLPRCPTFENWDDPIAEGYASHLAVDRTGYRYTFRPDNLHVRDLP-
-ELTKDNMRVWRDRIFDAATSCSVL--RENG---SFVKICRTR-------FYGGLNILGN
LIESNLRSINRMFYGNIHCYAHVIAARVTDPDGKYGQGNGVMYDVATSARDPLFYQWHKF
LDHFFYEHLTKLPTNHLFHLQ--NPDVSITNLEIISN-----------------------
------------------------------GRKNEIHTFWENDIMEISKGHSFTLNS-DA
KVKIQHLQHEKFEIHLTVQNDKGEDTDLFVRIFLLPLEDEESHELSLEEMVRMAVDIEKR
VIPAKPGSNDI-VISS--RSIGAPANKFFGSFEE-----------RYISEDCNFHSHCGW
PNYLLVPKGSSQGTPFAFVVMLTLAEDDFTPNM------------DDTC--FC--ADSWS
HCGSLFIQYPENVEMGFPFQPIIECT--KEEFF--A--LPNIAKQEVIIKFTGETKDSPL
VIQLE---------NDS-----------
```

Additional Figure A1. Multiple sequence alignment of myriapod hemocyanins and phenoloxidases in FASTA format. See Additional Table A2 for abbreviations of the proteins.
