# Supplementary material for: Diversity, evolution, and function of myriapod hemocyanins
Source: BMC Evol Biol. 2018 Jul 5;18:107. doi: 10.1186/s12862-018-1221-2 (PMC6034248; doi:10.1186/s12862-018-1221-2)
Supplement: Supplementary file 6 — Table S3. Expression of hemocyanin subunit mRNA in myriapods. (PDF 72 kb) [file 12862_2018_1221_MOESM6_ESM.pdf]

Additional Table A3. Expression of Hc subunit mRNA myriapods. The mRNA levels are given as RPKM (Reads Per Kilobase per Million mapped reads) values. See Table 1 for accession numbers.

Species/Gene	RPKM
Hanseniella sp.	
HanHcB	328.1
Scutigerella sp.	
ScuHcB	997.7
Cleidogona sp.	
CleHcBI1	37.6
CleHcBI2	259.5
CleHcBII	127.2
CleHcC	77.6
CleHcD	47.4
Abacion magnum	
AmaHcBI	68.2
AmaHcBII	84.4
AmaHcC	32.6
AmaHcD	47.1
Chamberlinius hualienensis	
ChuHcBI	1250.7
ChuHcBII	894.6
Polydesmus angustus	
PanHcBII1	169.1
PanHcBII2	26.7
Pseudopolydesmus sp.	
PseHcBI	195.5
PseHcBII	150.2
PseHcC	114.9
Prostemmiulus sp.	
ProHcBI	8134.2
ProHcBII	9777.6
ProHcC	2826.7
ProHcD	4849.2
Cambala annulata	
CanHcBI	416.0
CanHcBII	341.5
CanHcC	241.2
CanHcD	184.9

Species/Gene	RPKM
Scutigera coleoptrata	
ScoHcA	7484.2
ScoHcB	4967.9
ScoHcC	4532.0
ScoHcD	5731.1
ScoHcX	4.5
Scutigerina weberi	
SweHcA	478.5
SweHcB	343.4
SweHcC	560.0
SweHcD	522.3
Sphendononema guildingii	
SguHcA	1990.4
SguHcB	617.8
SguHcC	238.3
SguHcD	391.8
Alipes grandidieri	
AgrHcB	18.8
Cryptops hortensis	
ChoHcB	338.0
Newportia adisi	
NadHcA	179.2
NadHcB	260.0
NadHcD	134.2
Scolopendra mutilans	
SmuHcA	197.3
SmuHcB	169.2
SmuHcD	223.2
Scolopendropsis bahiensis	
SbaHcB	214.0
SbaHcD	59.2
Scolopendra dehaani	
SdeHcA	25.6
SdeHcB	48.5
Theatops spinicaudus	
TspHcB	6.0
